# Conduction‐Dominated Cryomesh for Organism Vitrification

**DOI:** 10.1002/advs.202303317

**Published:** 2023-11-28

**Authors:** Zongqi Guo, Nikolas Zuchowicz, Jessica Bouwmeester, Amey S. Joshi, Amanda L. Neisch, Kieran Smith, Jonathan Daly, Michael L. Etheridge, Erik B. Finger, Suhasa B. Kodandaramaiah, Thomas S. Hays, Mary Hagedorn, John C. Bischof

**Affiliations:** ^1^ Department of Mechanical Engineering University of Minnesota Minneapolis MN 55455 USA; ^2^ Hawaii Institute of Marine Biology University of Hawaii Kaneohe HI 96744 USA; ^3^ Smithsonian National Zoo and Conservation Biology Institute Front Royal VA 22630 USA; ^4^ Department of Genetics Cell Biology and Development University of Minnesota Minneapolis MN 55455 USA; ^5^ Taronga Conservation Society Australia Mosman New South Wales 2088 Australia; ^6^ School of Biological Earth and Environmental Sciences University of New South Wales Kensington New South Wales 2033 Australia; ^7^ Department of Surgery University of Minnesota Minneapolis MN 55455 USA; ^8^ Department of Biomedical Engineering University of Minnesota Minneapolis MN 55455 USA; ^9^ Graduate Program in Neuroscience University of Minnesota Minneapolis MN 55455 USA; ^10^ Institute for Engineering in Medicine University of Minnesota Minneapolis MN 55455 USA

**Keywords:** coral larvae, cryomesh vitrification, cryopreservation, Drosophila embryo, heat transfer, zebrafish embryo

## Abstract

Vitrification‐based cryopreservation is a promising approach to achieving long‐term storage of biological systems for maintaining biodiversity, healthcare, and sustainable food production. Using the “cryomesh” system achieves rapid cooling and rewarming of biomaterials, but further improvement in cooling rates is needed to increase biosystem viability and the ability to cryopreserve new biosystems. Improved cooling rates and viability are possible by enabling conductive cooling through cryomesh. Conduction‐dominated cryomesh improves cooling rates from twofold to tenfold (i.e., 0.24 to 1.2 × 10^5^ °C min^−1^) in a variety of biosystems. Higher thermal conductivity, smaller mesh wire diameter and pore size, and minimizing the nitrogen vapor barrier (e.g., vertical plunging in liquid nitrogen) are key parameters to achieving improved vitrification. Conduction‐dominated cryomesh successfully vitrifies coral larvae, *Drosophila* embryos, and zebrafish embryos with improved outcomes. Not only a theoretical foundation for improved vitrification in µm to mm biosystems but also the capability to scale up for biorepositories and/or agricultural, aquaculture, or scientific use are demonstrated.

## Graphical abstract

Vitrification‐based cryopreservation needs further improvement in cooling rates to increase biosystem viability and the ability to cryopreserve new biosystems. Higher thermal conductivity, smaller mesh wire diameter, and minimization of the nitrogen vapor barrier improve cooling rates up to 1.2 × 10^5^ °C min^−1^. Conduction‐dominated cryomesh successfully vitrifies coral larvae, *Drosophila* embryos, and zebrafish embryos with improved outcomes in a scalable way.

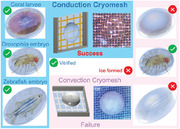

## 1 Introduction

Cryopreservation achieves long‐term storage by stopping the metabolic processes of biological systems at temperatures below the freezing point.^[^ ^1^, ^2^ ^]^ This is highly desirable for a range of biological systems for maintaining biodiversity,^[^ ^3^, ^4^ ^]^ healthcare,^[^ ^5^ ^]^ and food sustainability.^[^ ^6^ ^]^ One of the biggest sources of biological system damage during cryopreservation is ice formation.^[^ ^7^ ^]^ A cryoprotective agent (CPA) can be applied to avoid lethal ice formation by replacing intracellular water content in the biosystem and mitigating extracellular ice formation.^[^ ^2^, ^7^ ^]^ However, widely used CPAs and CPA cocktails can be toxic to cells and biosystems at higher CPA concentrations and temperatures.^[^ ^5^ ^]^ To reduce toxicity, CPA loading at lower temperatures and concentrations is typically employed. However, lower CPA concentrations require extremely high cooling and rewarming rates to avoid ice formation.^[^ ^4^, ^8^ ^]^

Cryopreservation in general can be achieved in the presence of controlled ice, or by vitrification, which seeks to avoid ice formation entirely. Slow freezing is one of the conventional methods to cryopreserve cells after stabilizing with low CPA concentration (e.g., 1.4 M DMSO (dimethyl sulfoxide), Table S1, Supporting Information).^[^ ^9^ ^]^ A cryovial is used to control the slow cooling rate (e.g., 1 °C min^−1^), which allows the growth of ice crystals outside of the cells. Cooling occurs slowly enough that the extracellular ice increases the CPA and solute concentration around cells, which leads to cellular dehydration, effectively increasing intracellular CPA/solute concentration and controlling intracellular ice formation. This is the basis for many conventional cryopreservation protocols, especially those used on cell lines (i.e., 1–2 M DMSO, 1 °C min^−1^ cooling; see ATCC, etc.). However, slow freezing often fails to achieve high viability in sensitive cell types such as T‐cells, stem cells, and hepatocytes,^[^ ^10^, ^11^, ^12^ ^]^ and is typically limited to smaller samples due to the variation in cooling rates experienced as sample size increases. Moreover, extreme osmotic stress and ice formation during the rewarming process remain critical challenges for slow freezing.^[^ ^13^ ^]^

**Figure 1:**
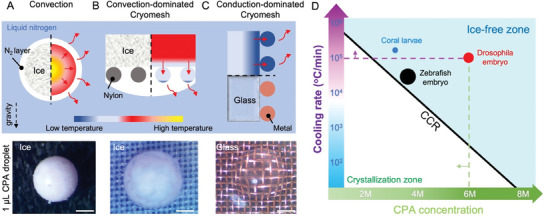
Conduction‐dominated cryomesh for cryopreservation. A) Droplets convectively cooled in LN_2_ show ice formation due to the nitrogen vapor layer that forms. B) The convection‐dominated cryomesh shows ice formation due to slower heat transfer through the nylon mesh. C) The conduction‐dominated cryomesh achieves vitrified droplets through enhanced heat transfer through the cryomesh and modified plunging techniques. CPA droplet volume is 1 µL, and the scale bars are 300 µm. Red solid arrows show the direction of heat transfer. The dashed arrow shows the gravity direction. Left/bottom of the dashed line shows the ice/glass state, right/top of the dashed line shows the thermal gradient. The temperature bar is not to scale. D) Conduction‐dominated cryomesh enables cooling rates above the critical cooling rate (CCR) for coral larvae, *Drosophila* embryo, and zebrafish embryo vitrification for a range of CPAs. The size of the dots (not to scale) represents biosystem size of 50 (blue), 500 (red), and 800 µm (black).

Vitrification or “ice‐free” cryopreservation at higher CPA concentrations and higher cooling and warming rates avoids both extracellular and intracellular ice formation by directly transitioning from liquid to glass state during cooling and then the reverse during warming.^[^ ^14^, ^15^ ^]^ Vitrification‐based cryopreservation has shown high viability for a wide range of biosystems.^[^ ^4^, ^5^, ^16^, ^17^, ^18^ ^]^ (Table S1, Supporting Information). Successful vitrification requires a cooling rate higher than the critical cooling rate (CCR), which is dictated by the choice of CPA and its concentration.^[^ ^19^, ^20^, ^21^ ^]^ Cooling at a rate slower than the CCR results in destructive ice formation. Low CPA concentrations require a higher CCR to achieve vitrification. Microliter droplets have been used for vitrification due to their relatively small thermal mass, which enables rapid cooling rates. However, when the cell‐laden droplet is directly immersed in liquid nitrogen (LN_2_),^[^ ^22^ ^]^ a nitrogen vapor layer forms around the droplet due to the boiling of LN_2_, which is also known as the “Leidenfrost effect”^[^ ^23^, ^24^ ^]^ (**Figure** **1**A). The low thermal conductivity and convective heat transfer coefficient reduce the droplet cooling rate (0.5 × 10^4^ °C min^−1^, Figure S1, Supporting Information), limiting the effective droplet size (<1 µL)^[^ ^18^, ^24^ ^]^ at typical CPA concentrations used in cell‐based cryopreservation.^[^ ^25^ ^]^ As a result, the convection droplet case has a lower cooling rate and demonstrates ice formation at larger droplet sizes (bottom, Figure 1A).

By directly printing or placing droplets on a substrate pre‐cooled to the temperature of LN_2_, a higher cooling rate can be achieved (2.1 × 10^4^ °C min^−1^).^[^ ^18^, ^26^, ^27^ ^]^ However, this droplet‐based method suffers from low throughput due to the need to process each droplet individually (e.g., in the µL min^−1^ range), which limits the scalability for clinical and industrial use (Table S1, Supporting Information). It should also be noted that the cryotop is another method commonly used for submillimeter droplet vitrification. Rates achieved with the cryotop are typically on the order of 2.3 × 10^4^ °C min^−1^ for a 0.1 µL droplet,^[^ ^28^ ^]^ which is much slower than evaluated here. This difference is due to the added thermal mass of the cryotop itself, which is a relatively large plastic substrate.

A promising alternative to droplet vitrification is the cryomesh vitrification^[^ ^29^, ^30^ ^]^ (Figure 1B and Figure S2, Supporting Information). Cryomesh can reach higher cooling rates by removing excess CPA around the biosystem through the mesh pores (Figure S3, Supporting Information) and minimizing the total thermal mass.^[^ ^31^ ^]^ Although mesh has previously been used to vitrify embryos,^[^ ^31^ ^]^ oocytes,^[^ ^30^ ^]^ and plants,^[^ ^32^ ^]^ to our knowledge, there are no design principles to choose the mesh appropriate for biosystems varying in size from micrometer to millimeter. Mesh materials in prior studies have been limited to nylon or stainless steel (**Table** **1**). Due to the low thermal conductivity of the nylon mesh and the vapor barrier caused by nitrogen bubbles, the mesh has a substantial temperature gradient and cannot reach the temperature of the LN_2_ any faster than by direct convective cooling of the biosystem. The entire system is dominated by the convection heat transfer condition, so we have termed the system “convection‐dominated cryomesh” (ConvD‐C). Ice still forms due to the lower cooling rate on the convection‐dominated cryomesh (bottom, Figure 1B). Meanwhile, mesh geometries (e.g., wire diameter and pore size) also vary across studies and do not appear to have been chosen systematically. Finally, while the cryomesh approach theoretically lends itself to scale‐up through increases in mesh area, there has been no study on the factors needed to enable this.

**Table 1:** Prior mesh‐based vitrification. Mesh size is described in terms of wire diameter and pore size, depending on what details were included.

Mesh material	Mesh size [µm]	CPA	CCR/CWR^a)^ [°C min^−1^]	Biosystem	Viability	Ref.
Nylon	N/A^b)^	15% EG + 15% DMSO + 17.1% (0.5 M) sucrose	1.3 × 10^3^/3.6 × 10^5^	Human embryo	98%	[29]
	75 pore	20% EG + 20% DMSO	4.5 × 10^3^/8.3 × 10^5^	Rabbit embryo	66%	[33]
	37–77 pore	15% EG + 15% DMSO + 17.1% (0.5 M) sucrose	1.3 × 10^3^/3.6 × 10^5^	Bovine mature oocytes	28–39%	[34]
	150 wire + 200 pore	39% EG + 9% sorbitol	5.5 × 10^1^/1.2 × 10^4^	*Drosophila* embryo	> 50%	[31]
	38 pore	22% EG + 22% DMSO	1.9 × 10^3^/2.6 × 10^5^	islets	> 87%	[5]
Stainless steel	224 wire + 400 pore	PVS2	3.4/1.5 × 10^2^	shoot tips	83%	[32]
	38 pore	75% VS55	1.5 × 10^3^/1.9 × 10^5^	kidney slice	91%^c)^	[35]
	38 pore	27% EG + 9% sorbitol	1.9 × 10^3^/6.8 × 10^5^	*Drosophila* embryo	60%^c)^	[35]

^a)^ Critical cooling rate (CCR) and critical rewarming rate (CWR) are calculated based on reference.^[^ ^21^ ^]^ EG (ethylene glycol) is simulated as PG (propylene glycol), DMSO is simulated as glycerol, sorbitol, and sucrose are simulated as trehalose;

^b)^ Not mentioned in the reference;

^c)^ Viability rate is increased due to ultrarapid rewarming through Joule heating. Not for vitrification comparison. VS55 is the name of CPA with 24% (3.1 M) DMSO (Me_2_SO) + 20% (3.1 M) Formamide + 17% (2.2 M) PG. PVS2 is the name of CPA with 15% EG + 15% DMSO + 30% glycerol + 13.7% (0.4 M) sucrose.

To address those challenges, we evaluated the basic heat transfer principles behind the cryomesh approach and conducted a systematic investigation of critical design parameters. A conduction‐dominated cryomesh is achieved by using high‐conductivity metal mesh (e.g., copper or stainless steel mesh) and optimizing the mesh geometry to allow rapid heat transfer from the biosystem through the mesh, which dramatically increases the cooling rate (Figure 1C and Figures S1 and S2, Supporting Information). Further, by modifying the method of plunge cooling to allow the release of the vapor barrier from the mesh (Figure S3, Supporting Information), the cooling of the biosystem is dominated by the conduction of heat through the cryomesh (Figure 1C). In this case, the heat of the biosystem can be released from the mesh to the LN_2_, where the mesh performs on par with a pre‐cooled substrate as in droplet printing methods. Thus, the cryomesh can reach high cooling rates, enabling droplet vitrification where convective methods have failed (bottom, Figure 1C and Figure S1, Supporting Information). By using modified plunging methods and enhancing the heat transfer in the mesh design, the cryomesh shows conduction‐dominated heat transfer performance. We have termed this “conduction‐dominated cryomesh” (CondD‐C). By varying the mesh wire size, pore size, and materials based on design principles, we successfully increased the cooling rate up to 10× over what was previously reported for convective cryomesh designs. Increased cooling rates enable the use of lower CPA concentrations, lowering the associated toxicity, which can help increase the viability of cryopreserved biosystems, and facilitating the cryopreservation of biosystems that have not yet been preserved due to susceptibility to CPA. The conduction cryomesh was then used to successfully vitrify three biosystems roughly ranging from micrometer to millimeter scale, namely coral larvae, *Drosophila* embryos, and zebrafish embryos with different CPA concentrations down to 3.5 M. This work demonstrates not only a theoretical foundation for improved vitrification in micrometer to millimeter biosystems but also the ability to scale up to create biorepositories and for agricultural, aquaculture, or scientific use.

## 2 Results and Discussion

### 2.1 Establishing Conditions to Achieve Conduction‐Dominated Cryomesh

The cryomeshes tested in this work are described in Figure S2, Supporting Information. We used width (*W*) and height (*H*) to define the geometry of the frame (3D‐printed PLA (polylactide)). If not specified otherwise, the frame to hold the mesh used in this study is *W* = 2 cm and *H* = 2 cm. We defined the cryomesh by its solid fraction (*Ф*) and wire diameter (*D*) with the unit of µm, where *Ф* = *D*/(*P+D*) and *P* is the pore size of the mesh. One of the main conduction‐dominated cryomeshes used in this study was the copper mesh (*Ф* = 0.5, *D* = 50 µm) with a frame size of 2 × 2 cm (*W* × *H*) (Figure S2A, Supporting Information). If not specified, the solid fraction is fixed as 0.5. Thus, the name of copper mesh (*Ф* = 0.5, *D* = 50 µm) is simplified as copper *D* = 50 µm. To further simplify the mesh definition, we also used critical length (*L*_c_) to compare different mesh geometries (Table S2, Supporting Information), which is a fundamental parameter often used for analysis in heat transfer (see detailed calculation in SI). Note that we chose to study wire diameter here due to the market availability with a round geometry. However, these results can also be thought of in terms of a representative characteristic length (e.g., volume divided by area), which can be more generally applied across different mesh filaments.

**Figure 2:**
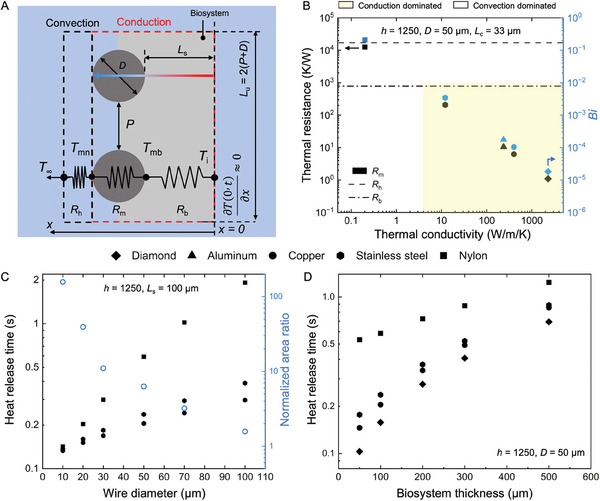
Design rationale of conduction‐dominated cryomesh. A) Heat transfer model of a cryomesh cooling in LN_2_. The right boundary is insulated to consider the conduction‐dominated limiting case. B) Thermal resistances of convection interface, mesh, and biosystem. A Biot number (*Bi*) value (Equation (5)) <0.01 indicates that the cryomesh is operating in a conduction‐dominated mode. Area shown in yellow indicates “conduction‐dominated” behavior. *L*_c_ is the critical length related to wire diameter and pore size (Table S2, Supporting Information). The thermal properties are included in Table S3, Supporting Information. C) Reduced mesh wire diameter, *D*, increases the contact area and decreases the heat release time. The heat release time *t* has an inverse correlation with the cooling rate (CR), that is, *t* ≈ Δ*T*/CR. D) Heat release time increases with increase of biosystem thickness. A conductive cryomesh reduces the heat release time for a thick biosystem.

Since we are attempting to describe the conditions in which heat release from the biosystem is dominated by conduction through the cryomesh, we used an idealized model with an adiabatic condition on the biosystem focusing on heat transfer through the mesh (**Figure** **2**A). This limiting case allows the impact of cryomesh design principles (thermal conductivity, pore size, and mesh diameter) to be optimized for improved cryomesh heat transfer performance. Importantly, any additional effects of heat transfer from the biosystem directly to LN_2_ will only enhance the rates. For completeness, the case where the biosystem also loses heat by convection to the LN_2_ is included in the Supporting Information (Figure S4A, Supporting Information). Thus, the biosystem thickness affected by conduction heat transfer is fixed to compare different mesh parameters. There are two processes of heat release with an adiabatic condition on the biosystem: 1) conduction heat transfer inside the mesh and biosystem and 2) convective heat transfer, which releases heat from the mesh to LN_2_. Since we are attempting to describe the conditions in which heat release from the biosystem is dominated by conduction through the mesh, for this analysis, heat release directly from the biosystem to LN_2_ is neglected. To describe the relative contributions to heat transfer, we analyzed a simplified thermal resistance model, which can be used to describe transient heat transfer in a model system. This model describes the heat flux (*q*3) during cooling from the cryomesh side, which will have a linear relation with the heat loss rate and thus cooling rate. We describe these conditions through a simple 1D thermal resistance model (Figure 2A), which is

(1) $$q^{\prime \prime }=\frac{\Delta T}{R_{h}+R_{m}+R_{b}}=\frac{T_{i}-T_{\infty }}{R_{h}+R_{m}+R_{b}}$$

where Δ*T* is the temperature difference between the initial temperature of biosystem and LN_2_, *R*_h_ is the external resistance of convection between the mesh and LN_2_, *R*_m_ is the internal conduction thermal resistance of the mesh, and *R*_b_ is the conduction thermal resistance of the biosystem. We assume CPA thermal properties are similar to water and the interfacial thermal resistance is considered between water and bulk material. Thus, the interfacial thermal resistance between cryomesh and CPA is ignored due to its small value (4–8 × 10^−9^ m^2^K W^−1^ << *R*_m_, 1 − 1000 m^2^K W^−1^).^[^ ^36^ ^]^ Then, we simplified those three thermal resistances as:

(2) $$R_{h}=\frac{1}{hA_{m}}$$

(3) $$R_{m}=\frac{D}{k_{m}A_{\mathrm{cm}}}$$

(4) $$R_{b}=\frac{L_{s}}{k_{b}A_{b}}$$

where *h* is the convection heat transfer coefficient between the LN_2_ and cryomesh, *A*_m_ is the contact area between LN_2_ and cryomesh, *A*_cm_ is the cross‐section area of the mesh, *D* is the mesh wire diameter, *k*_m_ is the thermal conductivity of the mesh, *k*_b_ is the thermal conductivity of the biosystem, *L*_s_ is the thickness of the biosystem, and *A*_b_ is the cross‐section area of the biosystem (see further details in SI). Thermal properties used for the calculation are included in Table S3, Supporting Information. Based on the equations (Equations (1)–(4), a small thermal resistance contributes to a high heat flux, which then equates to a higher cooling rate. Thus, there are three key parameters to achieve a higher heat loss by reducing the thermal resistances: 1) increasing the thermal conductivity of the mesh, 2) increasing the convection heat transfer coefficient with the cryomesh, and 3) reducing the mesh wire diameter. The thermal resistance of mesh, *R*_m_, decreases with an increase in *k*_m_ (Figure 2B). Assuming the convective coefficient^[^ ^37^ ^]^ and biosystem thickness, *L*_s_, are fixed (determined by LN_2_ plunge and biosystem geometry), increasing the thermal conductivity of the cryomesh is the first step to achieving a higher cooling rate. To achieve conduction‐dominated heat transfer, *R*_m_ should be smaller than *R*_h_ to ensure an effectively uniform temperature distribution throughout the mesh. Thus, the mesh has effectively the same temperature as LN_2_ throughout and can conductively cool the biosystem. Meanwhile, *R*_m_ should also be smaller than *R*_b_; otherwise, the mesh cannot transfer the heat of the biosystem and release it into the LN_2_. In that case, the major heat of the biosystem will be released from another side (away mesh side) into LN_2_ by convection heat transfer. To simplify this analysis, we used the Biot number (*Bi*) to identify conditions for determining CondD‐C behavior. In heat transfer, *Bi* is the typical metric used to describe the relative contributions of convection and conduction heat transfer, calculated as

(5) $$Bi=\frac{hL_{c}}{k_{m}}$$

where *L*_c_ = *V*/*A*_m_, which is the characteristic length scale of the conducting body with *V* equal to the volume of the mesh. *Bi* decreases with an increase of *k*_m_, following the same trend as *R*_m_ (Figure 2B). *Bi* also increases with *h*, which subsequently produces a nonuniform temperature (Figure S4, Supporting Information) across the conducting body if its thermal conductivity is not high enough. However, a high *h* is also required to achieve high cooling rates; thus, high thermal conductivity is required to maintain a uniform temperature of the mesh (Figure S4B, Supporting Information). Otherwise, the large thermal resistance of mesh conduction slows down the heat release (i.e., nylon mesh has a high *R*_m_). In contrast, a smaller *Bi* number (< 1) implies that conductive effects are greater than convective effects. When decreasing *h*, *Bi* is decreased with the *R*_h_ increase (Figure S4, Supporting Information). Thus, an *h* ≥ 2500 W m^−2^ K^−1^ is desired, which might be achieved by cooling in liquid nitrogen without a vapor barrier (Figure S5, Supporting Information).^[^ ^38^, ^39^ ^]^ Therefore, to achieve conduction‐dominated cryomesh behavior during cooling (yellow area, Figure 2B), we have defined that thermal conductivity of greater than ≈10 W m^−1^ K^−1^ is the minimum requirement to achieve *Bi* < 0.01 (conduction heat transfer dominates convection), assuming the highest expected convective conditions of *h* = 2500 W m^−2^ K^−1^ and mesh filament diameter of *D* = 50 µm (see further discussion on filament diameter below). Thus, for better performance with *h* varying from 250 to 2500 W m^−2^ K^−1^, the mesh thermal conductivity is recommended to be greater than 10 W m^−1^ K^−1^, with *D* < 50 µm to achieve *Bi* < 0.01 (i.e., “conduction‐dominated” behavior).

To further emphasize the advantages of the conduction‐dominated cryomesh, we calculated the heat release time based on several representative materials with a range of thermal conductivities, such as nylon, stainless steel, aluminum, copper, and diamond (Figure S6, Supporting Information). The model is simplified and shown in Supporting Information. The heat release time *t* has an inverse correlation with the cooling rate (CR), that is, *t* ≈ Δ*T*/CR. The increased thermal conductivity reduces the heat release time of the mesh, as expected. Diamond has the highest thermal conductivity at 2300 W m^−1^ K^−1^, with the lowest *Bi* number of 1.8 × 10^−5^ and a heat release time of 3.5 × 10^−5^ s, which is 0.008% of the nylon mesh. Meanwhile, more practically accessible materials such as copper and stainless steel have heat release times of 0.089% and 3.5%, respectively, of the heat release time of nylon mesh. To emphasize practical use, we focus on copper and stainless steel in further cryomesh demonstrations. The convection heat release time is mainly determined by the biosystem (Figure S6A, Supporting Information). As the conductivity of the biosystem is much smaller than that of the mesh (assumed to be 1.6 W m^−1^ K^−1^, representative of typical vitrified materials^[^ ^18^ ^]^), we assumed the heat of the biosystem has no effect on *t*_m_. The thermally conductive mesh can transfer the heat instantly (relative to the timescales of biosystem cooling) from the biosystem into LN_2_. Thus, the faster the mesh reaches the LN_2_ temperature (e.g., copper *t*_m_ < 1 × 10^−3^ s), the sooner the biosystem is cooled, and the mesh essentially maintains this temperature throughout cooling.

### 2.2 Model Prediction of Optimized Cryomesh System Design for Increased Cooling Rates

Besides increasing the convection heat transfer coefficient, the cooling rate can be further enhanced by 1) reducing the thermal resistance of the mesh, *R*_m_, and 2) reducing the thermal resistance of the biosystem, *R*_b_, based on the mesh geometry. With a fixed mesh material, the mesh heat release time (and thus cooling rate) can be further improved through optimization of the solid fraction *Ф* (≈*A*_cm_, Equation 3) and wire diameter *D*. The heat release time shows that the lowest values occur in the range of *Ф* = 0.5–0.66 (Figure S7, Supporting Information). While there is an optimal point in this range, the difference within the range is less than 10%, so values within this range can be used for practical optimization. The exact optimum depends on biosystem size and could be optimized for specific applications if desired. We further investigated the effect of mesh wire diameter on heat release time and cooling rate (Figure 2C and Figure S8, Supporting Information), understanding that this is a simple way to investigate the effects of the mesh surface area relative to mesh volume (i.e., thermal mass). For this case, the biosystem thickness is assumed to be 100 µm with an *h* of 1250 W m^−2^ K^−1^.^[^ ^37^ ^]^ The heat release time decreases with the decrease in the wire diameter (Figure 2C). For the same cryomesh critical thickness (i.e., same wire diameter and solid fraction), the copper mesh has a lower heat release time than the stainless steel mesh due to its higher thermal conductivity. Additionally, the total thermal mass of the cryomesh (impacted by wire diameter and pore size) is a critical factor. We found that a stainless steel mesh with a wire diameter of 30 µm (the finest commercially available mesh identified) had a smaller heat release time (0.18 s) than the larger wire diameter 50 µm copper mesh (0.21 s) (Figure 2C). Even though the thermal conductivity of stainless steel is smaller than copper, the smaller wire diameter reduces total thermal mass and thermal resistance (Equation 3). Thus, reducing the wire diameter reduces the heat release time and increases the cooling rate. Meanwhile, the surface contact area of *D* = 30 µm mesh is 2.8× as large as a mesh with *D* = 50 µm, assuming solid fraction is held constant (0.5). The increased contact area contributes to greater heat transfer from the biosystem by increasing the area of heat release. Thus, to further enhance the cooling rate, a smaller wire diameter is desired to increase the contact area between the mesh and biosystem. However, note that the nylon mesh is still in a convection‐dominated cooling process even at small filament diameters (i.e., nylon mesh will experience a substantial temperature difference across the mesh thickness). Even though a small wire diameter can decrease the heat release time, the slower rate of heat transfer through mesh mitigates the benefits of reduced wire diameter on thermal mass for meshes with low thermal conductivity (i.e., nylon). Thus, the reduction of mesh wire diameter and increased contact area enhance the cooling rate for conduction‐dominated cryomeshes. This trend will continue until reaching a small pore size (<5 µm) and small wire diameter (<20 µm). The pinning force of CPA transferring through pores increases with the decrease of mesh pore size and increase of liquid surface tension, which reduces proper wicking of the CPA.^[^ ^40^, ^41^ ^]^ Thus, small pore size nullifies the ability to wick away liquid thermal mass from the biosystem, which reduces the cooling and rewarming rate.^[^ ^31^ ^]^ Meanwhile, the wire tensile strength reduces with the reduction of wire diameter leading to easy breakage of the wire.^[^ ^42^ ^]^ Out of the commercially available meshes, we chose to evaluate a stainless steel mesh with *D* = 30 µm for further testing on the ability to vitrify a variety of biosystems (Figure S8, Supporting Information). With the same dimension of mesh (i.e., same wire diameter), higher thermal conductivity would contribute to a high cooling rate.

Biosystem thickness also affects the heat release time of the entire system during cooling (Figure 2D and Figure S9, Supporting Information). The increased thickness increases the total thermal mass and time required to conduct heat across the biosystem and so shows a longer heat release time for the system. The nylon mesh shows a longer heat release time with a 50 µm thickness biosystem, which is 2.6× and 2.0× that of the copper and stainless steel mesh, respectively. As predicted by the model, the diamond, with the highest thermal conductivity and lowest *Bi*, provides the fastest heat release times. For a biosystem with a thickness of 50 µm, the diamond has the shortest heat release time of 0.1 s, which is 70% that of copper. Compare this to stainless steel, which has a 21% longer heat release time than copper mesh. The heat release time of a thicker biosystem shows less variation with mesh size than a smaller biosystem (i.e., 50 µm), as the heat transfer inside the larger biosystem has a greater impact on the heat release time. Thus, CondD‐C is desired to achieve a high cooling rate for a wide range of thicknesses of biosystems. However, with increased biosystem thickness, the difference in heat release time between copper and stainless steel is reduced to only 3% for a biosystem of 500 µm thickness. The low thermal conductivity and high thermal mass of the biosystem lead to high heat transfer times inside the biosystem.

As a general design range to push forward the cooling rate, CondD‐C should have *Bi* < 0.01, which has detailed parameters of *k*_m_ ≥ 10 W m^−1^ K^−1^, *D* ≤ 50 µm, and 0.65 ≥ *Ф* ≥ 0.5. With the consideration of easy access and costs, copper and stainless steel (with appropriate choices in mesh design) are practical means to achieve conduction‐dominated cryomesh behavior, but materials such as diamond could be used to achieve theoretically optimal cooling behavior. The combination of more than one type of metal, such as copper with gold coating or CVD diamond coating, could allow for achieving higher rates than copper or stainless steel alone due to the increased contact area and reduced thermal resistance.^[^ ^43^ ^]^

### 2.3 Experimental Validation of Cooling Rate on the Conduction‐Dominated Cryomesh

**Figure 3:**
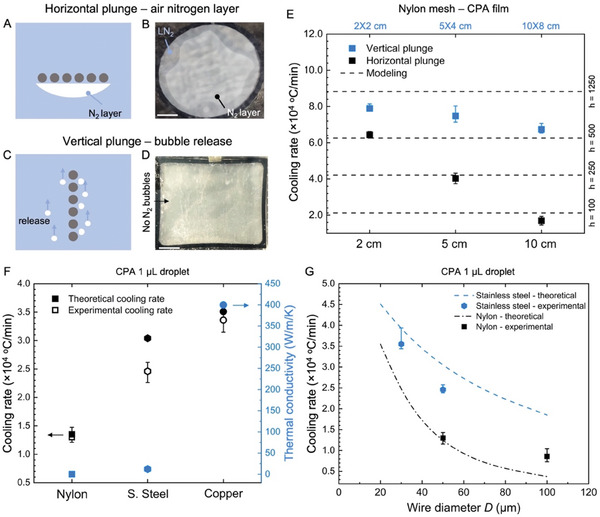
Experimental validation of cooling rate varying as a function of mesh design and plunging. A) Schematic and B) camera image of horizontal LN_2_ plunge with trapped bubbles. C) Schematic and D)camera image of vertical LN_2_ plunge with release of vapor layer. E) Measured cooling rates for vertical and horizontal plunge varying with mesh sizes and material at the geometric center of the mesh. The model suggests a lower effective heat transfer coefficient on the horizontal plunge due to trapped bubbles. F) Measured cooling rate of nylon, stainless steel, and copper compared with thermal conductivity. The higher thermal conductivity of copper produces a higher cooling rate. G) Experimental and theoretical cooling rate of different mesh wire diameters. The scale bar in B and D is 1 cm.

We next directly measured the cooling rate of the conduction‐dominated cryomesh based on varying materials and wire diameters. This first required an evaluation of the impact of the plunge methods (**Figure** **3**A–D, Figure S10 and Movie S1, Supporting Information). When a warm substrate is submerged into LN_2_, a rapid phase change in LN_2_ occurs and creates a vapor layer around the substrate, a phenomenon known as the Leidenfrost effect (film boiling region, Figure S5, Supporting Information). If the substrate is plunged into LN_2_ with a horizontal orientation, the nitrogen vapor is easily trapped underneath the mesh, forming a thick insulating layer (Figure 3A,B and Figure S10A, Supporting Information). This vapor layer has a lower thermal conductivity and a higher temperature than the LN_2_. This effectively reduces the convection heat transfer coefficient between the mesh and liquid nitrogen. Thus, the cooling rate is reduced. Due to larger thermal mass, a larger mesh surface area generates more nitrogen vapor bubbles, which are trapped by the mesh, building up a thicker gas layer than smaller meshes, especially at the center of the mesh (Figure S10, Supporting Information). Therefore, methods are required to reduce or eliminate this vapor barrier to achieve the fastest possible cooling rates, especially when cryomesh is scaled up.

One simple and effective method for reducing Leidenfrost on the cryomesh is to increase *h* by a vertical plunge. Vertical plunging allows nitrogen bubbles to rapidly form and release from the mesh, greatly reducing the vapor barrier around the mesh (Figure 3C,D, and Figure S10B, Supporting Information). In theory, this can be similarly accomplished by agitating the flow of the LN_2_, by mechanical motion of the cryomesh, or by modifying the mesh surface to facilitate boiling LN_2_ vapor release. It also provides opportunities to tune the LN_2_ boiling curve (e.g., increase critical heat flux and its subcooling, Figure S5, Supporting Information) to reach a higher heat transfer^[^ ^44^ ^]^ and for other cryogens.^[^ ^38^, ^45^ ^]^ To quantitatively study the effect of different plunge methods, we compared the cooling rate between the vertical and horizontal plunge methods on a bare mesh (i.e., no CPA loading, Figure S11), then CPA film‐loaded mesh (Figure 3E), and 1 µL CPA droplet‐loaded mesh (Figure S12, Supporting Information). If not specified, the cooling rate was measured at the geometric center of the mesh. The thin CPA film (with a thickness of around 2 µm) simulates a small biosystem thermal load, while a 1 µL CPA droplet (*L*_C_ ≈ 500 µm) simulates the largest biosystem tested later in this study. The cooling rates of the different biosystem sizes show a similar trend (Figure S13, Supporting Information). The cooling rate showed significant variation for the horizontal plunge case from the center to the edge (2.8× higher at the edge than the center) versus the rate observed for the vertical plunge method (Figures S11 and S12, Supporting Information). The horizontal plunge shows a sharp decrease in cooling rate from 6.4 × 10^4^ °C min^−1^ at 2 × 2 cm to 1.7 × 10^4^ °C min^−1^ at 10 × 8 cm. The vertical plunge presents a marginal decrease (18%) in the average cooling rate as the mesh size increases (i.e., 10 × 8 cm), which is significantly smaller than the decrease in the average cooling rate of the horizontal plunge (73%). Meanwhile, the vertical plunge achieves an average cooling rate across the mesh of 7.8 × 10^4^ °C min^−1^ versus 6.4 × 10^4^ °C min^−1^ with the increase of the mesh size from 2 × 2 cm to 10 × 8 cm, showing a much more uniform cooling than horizontal plunge (Figures S12 and S14, Supporting Information).

Thus, we also studied the uniformity of cooling across small to larger mesh areas for further scale‐up designs (Figure S14, Supporting Information). A small‐size mesh (i.e., *H* = 2 cm) has uniform cooling along the plunge direction, showing a difference of less than 6% (Figure S14B, Supporting Information). For the vertical plunge of 5 cm × 4 cm mesh, we measured 5 points to test the uniformity of the cooling rate on copper and nylon mesh (Figure S14C, Supporting Information). The CondD‐C demonstrated uniformity across the area within 6% (difference between highest and lowest value), while the ConvD‐C demonstrated nonuniformity with variation up to 34% (nylon mesh 4‐µL droplet, Figure S14C, Supporting Information). Interestingly, the cooling uniformity is only related to the height (*H*) of the cryomesh during the vertical plunge (Figure S14D, Supporting Information). With an increase of *H* from 2 to 8 cm, the temperature differences between the top and bottom of the cryomesh increase from 1% to 26%. When the width (*W*) of the cryomesh changes from 5 to 15 cm with a fixed height (*H*), the cooling rate of the bottom slightly decreases by 1%, and the temperature differences between the top and bottom of the cryomesh increase from 7% to 9%, respectively. The height (*H*) of cryomesh has a limited effect on cooling uniformity (Figure S14D, Supporting Information). During the vertical plunge, bubbles (vapor nitrogen) still are generated due to heat release from the cryomesh and the biosystem. The bubble rises from the bottom of the cryomesh toward the top due to buoyancy force and coalesces with other bubbles, potentially forming a thick vapor layer around the cryomesh similar to flow boiling.^[^ ^46^ ^]^ Thus, with the increased height of cryomesh, the cooling rate is reduced due to the reduced convection heat transfer of the bubble layer. For scaling to a larger mesh area, the width can be increased with minimal impact on the rate or uniformity of cooling, while height needs to be more carefully designed within the requirements of a specific cooling application.

Using the heat transfer model, we varied the heat transfer coefficient to fit the experimental cooling rate (Figure 3E) and determine the effective heat transfer coefficients for our plunge cases. For a 10 cm circular mesh with a horizontal plunge, the simulated heat transfer coefficient is around 100 W m^−2^ K^−1^ due to the insulating nitrogen vapor layer trapped underneath the mesh (estimated using the heat transfer model (Equation (1)) to fit with the experimental cooling rate data). The vertical plunge increases the effective heat transfer coefficient by allowing the release of the nitrogen vapor bubbles, which is one key factor in achieving optimal cooling rates with the conduction‐dominated cryomesh. Here we simulated an effective heat transfer coefficient from 500 to 1250 W m^−2^ K^−1^ for the vertical plunge case (estimated using the heat transfer model (Equation (1)) to fit the experimental cooling rate data). Meanwhile, the vertical plunge achieves uniform cooling across the surface, which is essential for mesh scale‐up. To further enhance the bubble release, a general design principle is to apply a hydrophilic coating on the mesh.^[^ ^47^ ^]^ The hydrophilic coating (e.g., PEGylated coating^[^ ^48^ ^]^) allows the LN_2_ to wet the mesh more easily than a hydrophobic coating due to its high surface energy.^[^ ^49^ ^]^ Thus, the bubble has less contact area with the mesh and a reduced pinning force, which leads to a rapid release from the substrate.^[^ ^50^, ^51^ ^]^ Future studies will investigate the optimization of coating on cryomesh to enhance the bubble release, especially for enhanced convection (Table S4, Supporting Information). Similar to enhancing the critical heat flux (CHF) of boiling, previous studies have shown potential methods to reduce or eliminate vapor barriers, such as using hydrophilic coatings,^[^ ^49^ ^]^ nanostructures,^[^ ^52^ ^]^ or 3D geometries^[^ ^53^ ^]^ (Table S4, Supporting Information).

Once we had determined the optimal plunging conditions, we compared the cooling rate of several commercially available cryomesh designs with different wire diameters (Figure 3F,G and Figure S8, Supporting Information). The cooling rates of nylon, stainless steel (s. steel), and copper mesh with *D* = 50 µm and *Ф* = 0.5 were measured with a 1‐µL droplet with a concentration of 14 wt % EG + 14 wt % DMSO + RPMI (Figure 3F). The copper mesh showed the highest cooling rate of 3.4 × 10^4^ °C min^−1^, which is 1.4× and 2.6× that of stainless steel and nylon mesh with the same dimensions, respectively. For comparison, we also included the theoretical cooling rates, which show a trend similar to the experimental cooling rate, which increases with the thermal conductivity (Figure 3F and Figure S14B, Supporting Information). For this modeling, the heat transfer coefficient is a simple experimental fitting, 1250 W m^−2^ K^−1^ to match the experimental cooling rate (1‐µL droplet case). When the filament diameter decreases from 50 to 30 µm, for stainless steel, the experimental cooling rate increases by 2.5 to 3.6 × 10^4^ °C min^−1^ for the 1‐µL droplet (Figure 3G). As a result of lower thermal mass and higher contact area, a 30 µm diameter mesh shows a cooling rate similar to a copper mesh with a 50 µm wire diameter, as predicted in the modeling (Figure S8, Supporting Information). Note that the model predicts a slightly higher cooling rate, as the simplified fitting did not consider the dynamic change of the heat transfer coefficient during the cooling process, especially for large thermal mass heat releases. In this case, a large thermal mass continually releases more heat into the LN_2_, which generates bubbles more rapidly around the mesh and slightly lowers the convection heat transfer coefficient. The measured nylon mesh cooling rates are also slightly lower than the predicted values. When the diameter of nylon mesh increases to 100 µm, the low thermal conductivity of nylon severely limits heat transfer through the mesh. Thus, the nylon with *D* = 50 µm has a similar experimental cooling rate to nylon with *D* = 100 µm (Figure 3G). In this case, the major heat release from the biosystem is from direct convection between the biosystem and LN_2_, which was neglected in the model, as discussed above. Therefore, regardless of a diameter of 50 or 100 µm for nylon, the mesh serves mainly as a carrier holding biosystems during plunging into LN_2_ but does not participate significantly in heat transfer during cooling.

While the copper mesh demonstrated some of the fastest cooling rates, one potential concern with copper was toxicity with direct exposure to the biosystems. Copper is considered toxic to many biological systems with even short time exposure (less than 1 h) showing damage to the cell membrane and entering the cells if released as ions.^[^ ^54^, ^55^ ^]^ While copper mesh is a better physical choice based on conductivity alone, we wished to eliminate any concerns related to toxicity. Thus, we chose to conduct further testing in biological systems with stainless steel, which is well‐established as a biocompatible material to reduce toxicity. If not otherwise specified, for further application on biosystems, we chose a stainless steel mesh with *D* = 30 µm and *Ф* = 0.5, which had a similar cooling rate to copper *D* = 50 µm. Copper mesh was only used to compare the cooling rate and validate the modeling.

### 2.4 Considerations for Cryomesh Design for Rewarming

One of the benefits of the cryomesh approach is that rewarming is subject to very similar thermal physics to that already presented for cooling. In this case, rather than LN_2_, the vitrified cryomesh can be plunged into a rewarming (physiological solution) bath set to the desired temperature. The mechanisms of rewarming are still convection and conduction, as noted for cooling (see Figure 2A). Therefore, the same principles of cryomesh design optimization apply, with the important caveat that the Leidenfrost effect will not be present and therefore the heat transfer coefficient can be larger (with an expected range of 1250 to 5000 W m^−2^ K^−1^), especially if agitation or flow is induced.^[^ ^39^ ^]^ Using a 50 µm thick biosystem as a model and increasing the *h* accordingly, conductive mesh rewarming rates can increase up to 3.5 times, reaching a rewarming rate of 4.4 × 10^5^ °C min^−1^ compared with *h* from 1250 to 5000 W m^−2^ K^−1^. The nylon ConvD‐C has a lower conductivity, so its rewarming rate only increases by 10%, reaching 0.17 × 10^5^ °C min^−1^ from *h* = 1250 to 5000 W m^−2^ K^−1^. (Figure S15A, Supporting Information). This conduction‐dominated rewarming rate can also be enhanced in thicker biosystems and, as before, improved by mesh conductivity (Figure S15, Supporting Information). Meanwhile, the experimental rewarming rate of CondD‐C (stainless steel) is 2.6 × 10^5^ °C min^−1^, which is 15% lower than the model prediction but is 2.4 times that of ConvD‐C (nylon) (Figure S16, Supporting Information). Therefore, optimizing the CondD‐C design for improved vitrification will inherently improve cases of plunge‐based rewarming.

With the successfully optimized physics of cooling and rewarming with the cryomesh, the next sections will deal with application to important biosystems including coral larvae, *Drosophila* embryos, and zebrafish embryos.

### 2.5 Coral Larvae Cryopreservation

Cryopreservation of coral larvae is challenging due to their high sensitivity to chilling and CPA toxicity, which requires the use of CPA cocktails with lower overall concentrations than are typically used in the cryopreservation of aquatic species.^[^ ^4^ ^]^ Adult mushroom coral larvae (*Lobactis scutaria*) have been successfully cryopreserved with a recovery rate of 43% following vitrification in 1‐µL droplets containing 8–20 larvae per droplet on a cryotop using 3.5 M CPA with laser rewarming, achieving a simulated rewarming rate of 4.5 × 10^6^ °C min^−1^^[^ ^4^ ^]^ However, droplet‐based vitrification approaches are not readily suited to large‐scale coral restoration efforts because of their complexity, the need for extensive training, and the small number of larvae processed (100–300 each day). Thus a simple, scalable technology that focuses on efficient cryoprotectant loading and produces rapid cooling and rewarming is critically needed to support coral settlement and reef restoration.^[^ ^56^ ^]^ Here, we demonstrate that the conduction‐dominated cryomesh approach enables rapid vitrification and rewarming of coral larvae with a high survival rate and can readily be scaled to larger numbers through the use of larger or multiple cryomesh.

**Figure 4:**
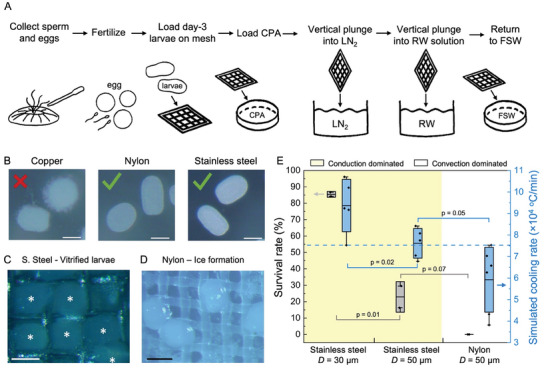
Vitrification of coral larvae. A) Schematic of vitrification protocol for coral larvae (*Lobactis scutaria*) with vertical plunge. B) Microscope images of coral larvae unloaded from copper, nylon, and stainless steel meshes. Copper demonstrated toxicity to the coral larvae and was not used. C) Microscope images of coral larvae after vitrification on stainless steel with *D* = 50 µm and *Ф* = 0.33. Vitrified larvae are transparent. D) Microscope images of coral larvae after cryopreservation process on nylon mesh with *D* = 50 µm and *Ф* = 0.5. Larvae have ice formation, showing white color. E) Survival rate and simulated cooling rate of stainless steel and nylon mesh with vertical plunge. The conduction‐dominated cryomesh (yellow‐colored area) has a higher survival rate due to a higher cooling rate with *D =* 30 µm and *Ф =* 0.5 being the best. Gray boxes represent standard deviation of survival rates. Blue boxes represent standard deviation of the simulated cooling rate. Horizontal dash line is estimated as the threshold cooling rate higher than CCR. The scale bars are 100 µm.

The steps involved in the cryopreservation of coral larvae utilizing CondD‐C are presented in **Figure** **4**A. Larvae from the mushroom coral *L. scutaria* were produced during annual spawning in Hawaii as previously described.^[^ ^4^ ^]^ Day‐3 coral larvae were transferred from seawater to the CondD‐C and immersed in CPA solution (10% v/v PG + 5% v/v DMSO + 1 M trehalose) for 2 min, followed immediately by wicking of excess CPA from the mesh and vertical plunging into LN_2_. At this point, the coral larvae could be placed in storage; however, for this demonstration, the CondD‐C mesh with larvae was immediately rewarmed by vertically plunging into rewarming solution (RW) and filtered seawater (FSW) (see further details in Experimental Section).

In the process of conducting experiments, we found that copper mesh exposure was toxic to coral larvae and therefore we did not use it in any of the following experiments except to compare its achievable cooling rate (Figure 4B). Thus, we vitrified the coral larvae on nylon mesh (*D* = 50 µm, *Ф* = 0.5) and stainless steel mesh (*D* = 30 µm, *Ф* = 0.5 and *D* = 50 µm, *Ф* = 0.33). Vitrification proceeded as described earlier and in Figure 4A. We used a stereomicroscope to assess the vitrification of the coral larvae. Ice can be visualized as white clouding, while vitrified larvae remain transparent. The stainless steel cryomesh showed good vitrification (Figure 4C), but larvae on the nylon mesh appeared to be entirely crystallized (Figure 4D). Based on the experimental data, stainless steel with *D* = 30 µm and *Ф* = 0.5 can achieve the best outcomes with larvae cooling rates in excess of 1.2 × 10^5^ °C min^−1^. This compares to cooling rates for the nylon mesh which are expected to be only 0.78 × 10^5^ °C min^−1^ (Figure S13, Supporting Information).

After establishing successful vitrification, we quantified the survival rate of coral larvae rewarmed on the stainless steel and nylon mesh (Figure 4E). Here, survival is defined as the resumption of swimming by 2 h post‐thaw (see Methods for further details). To assess proof‐of‐concept cryopreservation success in coral larvae we used the coral swimming test. This test is the easiest and fastest method to assess the viability.^[^ ^4^ ^]^ The stainless steel cryomesh with *D* = 30 µm achieved the highest survival rate at 85% (n = 200 coral larvae per test), while the nylon‐based cryomesh produced a 0% survival rate. The high survival rate achieved by the stainless steel cryomesh with smaller filaments was attributed to the high cooling rate that was higher than the CCR of CPA for coral larvae (Figure 4E and Table S5, Supporting Information). Due to practical constraints, we were not able to directly measure the cooling rates of the coral larvae. To estimate these rates, we used PE (polyethylene) particles with a diameter of 100 µm (Cospheric LLC) loaded with CPA to simulate the vitrification of coral larvae and measured the cooling rate. The stainless steel mesh with *D* = 30 µm achieved the highest average cooling rate of 9.4 ± 1.2 × 10^4^ °C min^−1^, which was 1.2× and 1.6× of stainless steel with *D* = 50 µm and the nylon cryomesh, respectively. The smaller wire diameter contributed to a higher cooling rate and a higher survival rate than the thicker wire diameter. The calculated CCR of CPA for coral larvae was 1.3 × 10^3^ °C min^−1^, which was at least one order smaller than the achievable cooling rate on cryomesh (Figure 4E and Table S5, Supporting Information).^[^ ^21^ ^]^ However, even though nylon mesh achieved a cooling rate higher than CCR, no coral larvae were vitrified (Figure 4D). The CPA concentration inside coral larvae might not be able to reach the designed CPA concentration.^[^ ^57^ ^]^ Thus, the CCR required for coral larvae is higher than the cooling rate of nylon mesh showing unvitrified larvae. Instead of using CCR, the threshold cooling rate for coral cryopreservation was estimated as 7.5 × 10^4^ °C min^−1^ based on the experimental result of survival rate (dashed line, Figure 4E), which was one order higher than the CCR of coral larvae CPA. The range of cooling rates achieved for each of the mesh cases directly correlated to the observed survival (Figure 4E). As discussed earlier, the principles leading to increased cooling rates will also imply an increased rewarming rate, so some of the differences in survival on the same CondD‐C may also be attributed to the CPA concentration differences between coral larvae. The survival rate difference between different CondD‐C may be attributed to the ability of CondD‐C to achieve the CWR of the CPA or the uniformity of cooling within larvae.

The cryopreservation efficiency is improved by using CondD‐C to achieve high viability and uniform cooling and rewarming with a large number of individual biosystems (i.e., larvae or embryo) loaded (number ≥100). As one example, to achieve 100 000 alive coral larvae after cryopreservation, the total time of the laser‐associated method^[^ ^4^ ^]^ is 456× longer than the cryomesh method (Table S6, Supporting Information). The next step for this mesh technology is to try cryopreserving coral larvae from other species that are known to settle well on artificial substrates. This will then allow us to conduct more comprehensive behavioral and gene expression analyses on the coral to test functionality after cryopreservation.

### 2.6 *Drosophila* Embryo Cryopreservation

To further investigate biosystem vitrification using the CondD‐C, we attempted to cryopreserve *Drosophila* embryos, which have a greater thickness than the coral larvae above. Previous work has cryopreserved *Drosophila* embryos on cryomesh with a CPA concentration of 27% followed by convection rewarming, demonstrating average hatching and survival‐to‐adulthood rates of ≈10–12%.^[^ ^31^, ^35^ ^]^ Joule heating has been applied to rewarm vitrified *Drosophila* embryos and improved average hatching and adult rates to 60.8% and 41.3%, respectively.^[^ ^35^ ^]^ Therefore, to further improve hatching and adult rates during cryopreservation, higher vitrification rates or lower CPA concentrations are desired. Here, we demonstrated the ability of the CondD‐C approach to improve the vitrification of *Drosophila* embryos with low CPA concentrations.

**Figure 5:**
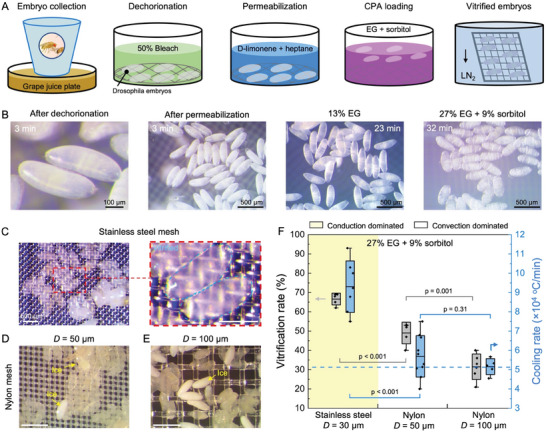
Vitrification of *Drosophila* embryos. A) Schematic of vitrification protocol for *Drosophila* embryos loaded with 27% EG and 9% sorbitol with vertical plunge. B) Microscope images of *Drosophila* embryos during loading process. C) Microscope images of *Drosophila* embryos after vitrification on stainless steel with *D* = 50 µm and *Ф* = 0.33. The image on the right is the zoomed‐in view with a scale bar of 250 µm. D) Microscope images of *Drosophila* embryos after vitrification on nylon mesh with *D* = 50 µm and *Ф* = 0.5. E) Microscope images of *Drosophila* embryos after vitrification on nylon mesh with *D* = 100 µm and *Ф* = 0.33. Vitrified larvae are transparent. Larvae with ice formation are opaque and white in color. F) Vitrification rate and cooling rate of stainless steel and nylon mesh with vertical plunge. The conduction‐dominated cryomesh (colored area) has a higher vitrification rate due to a higher cooling rate. Gray boxes represent standard deviation of survival rates. Blue boxes represent standard deviation of simulated cooling rate. Horizontal dashed line is the estimated threshold cooling rate, which is higher than CCR for *Drosophila* embryo. The scale bars are 500 µm for (C–E).

The steps involved in the cryopreservation of *Drosophila* embryos utilizing CondD‐C are presented in **Figure** **5**A (further details in Experimental Section). We used a derivative of the wildtype stock *w* ^1118^ called M2.^[^ ^31^ ^]^ The *Drosophila* embryos were collected on a grape juice plate and allowed to develop for 22 h at 20 °C. The embryos were dechorionated with 50% bleach and permeabilized with D‐limonene and heptane (Figure 5A,B). Cryoprotective agent step loading was followed with concentrations of 13% EG and 27% EG + 9% sorbitol, successively, used by the previous study as the standard protocol.^[^ ^35^ ^]^ The embryos’ shrinkage and crenation (wrinkling) showed successful dehydration of the embryos (32 min, Figure 5B). *Drosophila* embryos were placed in a nylon mesh basket for all loading and dehydration steps. The embryos were then transferred to ConvD‐C or CondD‐C, excess CPA was wicked away, and they were plunged vertically into LN_2_. We tested embryo vitrification on the stainless steel mesh and two nylon meshes with different filament diameters (Figure 5C–E). The 100 µm diameter nylon was used to provide a direct comparison to previous studies,^[^ ^31^ ^]^ and the 50 µm diameter nylon mesh was used to provide a closer comparison to the 30 µm stainless steel mesh. We used a microscope (Amscope) with a camera (MU1000) to visualize the vitrified *Drosophila* embryos.^[^ ^35^ ^]^ The number of *Drosophila* embryos used for each individual test loaded on a cryomesh was *n* = 200–400 with a total number of ≈10 000. The vitrified embryos were transparent, with the mesh visible underneath the embryos (right, Figure 5C), while embryos with internal ice showed opaque white color (Figure 5D,E). We calculated the vitrification rate based on representative microscope images from each run (Figure 5C–E). On the stainless steel mesh (*D* = 30 µm, *Ф* = 0.5), the majority of the embryos were vitrified, achieving a high vitrification rate of 66 ± 3%, which was higher than the nylon mesh with *D* = 50 µm (48 ± 5%) and *D* = 100 µm (30 ± 7%) (Figure 5C,F). Stainless steel mesh achieved the highest average cooling rate of 8.8 ± 1.4 × 10^4^ °C min^−1^, which was 1.6× and 1.8× that of the *D* = 50 µm and *D* = 100 µm nylon cryomesh, respectively. Based on the experimental results of vitrification rate, a cooling rate of 5.1 × 10^4^ °C min^−1^ was estimated as the threshold cooling rate for *Drosophila* embryos loaded with 27% EG and 9% sorbitol to be vitrified (dashed line, Figure 5F), which is higher than the required CCR for *Drosophila* embryos (Table 1 and Figure S5, Supporting Information). We believe the clustered embryos on the CondD‐C led to variability in cooling rates and a vitrification rate lower than 100%. The clustered embryos effectively increased the biosystem thermal resistance with increased thickness (*R*_b_, Equation 4), thereby decreasing the cooling rate. Interestingly, it was observed that a monolayer of *Drosophila* embryos on the cryomesh showed better vitrification than clustered embryos (right bottom, Figure 5C), close to a 100% vitrification rate. Meanwhile, a high vitrification rate was achieved on CondD‐C due to its high cooling rate, which also had a direct relationship with the hatch rate (Figure S17, Supporting Information). The previous study achieved a high average hatch rate (up to 52.9%) on a nylon mesh using a higher CPA concentration (39% EG + 9% sorbitol) which permitted lower CCR and CWR requirements.^[^ ^31^ ^]^ However, when decreasing the CPA concentration to 27% EG + 9% sorbitol, there was low hatching (4.4 ± 3%) of *Drosophila* embryos on ConvD‐C (nylon, *D* = 100 µm) due to a low cooling rate (Figure S17, Supporting Information). Meanwhile, hatching rates using the lower CPA concentration on the improved CondD‐C demonstrated here were up to 24 ± 6.6%, which was 3.3× that of an improved nylon mesh (with *D* = 50 µm) and 5.4× the hatching rate with prior use of the stainless steel mesh with the same CPA concentration.^[^ ^35^ ^]^ We also did additional morphological analysis of the *Drosophila* embryo after rewarming and hatching with CondD‐C (Figure S18, Supporting Information). The control embryo and larvae morphology showed no change (number of biosystems with morphology change <1%) compared to those after vitrification and rewarming.

With a high cooling rate achieved on CondD‐C, we demonstrated a method to lower the required CPA concentration while maintaining vitrification, rewarming without crystallization, and high hatch rates. This will be critical for expanding use to biosystems that are more susceptible to CPA toxicity. Although the enhanced vitrification rates, rewarming rates, and hatch rates were achieved by CondD‐C optimization, it is important to note that even faster rates of rewarming, and possibly even better hatch rates, may be achieved by Joule heating to minimize or avoid ice formation during rewarming.^[^ ^35^ ^]^

### 2.7 Zebrafish Embryo Cryopreservation

With the successful cryopreservation of small organisms, the upper size limit of organisms to which the conduction‐dominated cryomesh could be applied was tested with zebrafish embryos (diameter = 800 µm). The first successful protocol for zebrafish embryo vitrification employed a cryotop‐like device made with a polypropylene strip.^[^ ^58^, ^59^, ^60^ ^]^ However, such a protocol can vitrify one zebrafish embryo on cryo‐top at a time, considerably limiting the throughput of cryopreservation. With the previous protocol, a well‐trained operator can only vitrify 10–15 embryos in an hour. Further, as noted earlier, cooling rates on the cryotop are considerably lower than those achievable with the CondD‐C (Table S1, Supporting Information). Faster cooling rates can also enable lower CPA concentrations to be used, which could further increase viability.^[^ ^59^ ^]^ Thus, a substrate with a high cooling rate that can vitrify large quantities of zebrafish embryos is desirable. Here, we demonstrate the ability of the CondD‐C approach to achieve scalable, high‐throughput vitrification of zebrafish embryos.

**Figure 6:**
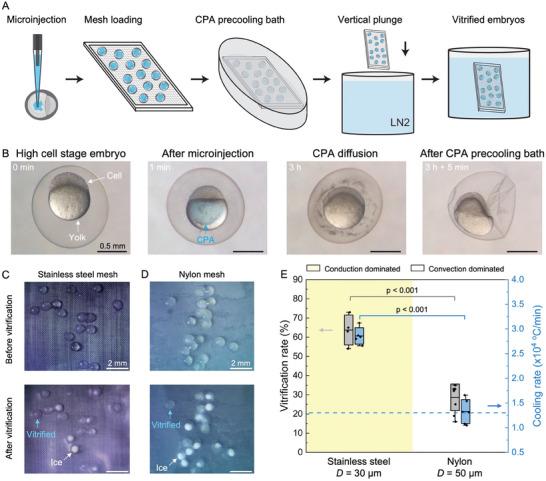
Vitrification of zebrafish embryos. A) Schematic of zebrafish embryo vitrification protocol. B) Microscope images of zebrafish embryos at different stages of the protocol. The scale bar is 0.5 mm. C) Microscope images of zebrafish embryos before and after vitrification on stainless steel with *D* = 30 µm and *Ф* = 0.5. Embryos showing ice formation are white in color, while the vitrified embryos are transparent. D) Microscope images of zebrafish embryos before and after vitrification on nylon with *D* = 50 µm and *Ф* = 0.5. The scale bars for (C) and (D) are 2 mm. E) Vitrification rate and experimental cooling rate of stainless steel and nylon mesh with vertical plunge. Yellow‐colored area shows conduction‐dominated cryomesh cooling. Gray boxes represent standard deviation of survival rates. Blue boxes represent standard deviation of simulated cooling rate. Horizontal dash line is the estimated threshold cooling rate, which is higher than the CCR of zebrafish embryos. The conduction‐dominated cryomesh has a higher vitrification rate due to a higher cooling rate.

The cryopreservation of zebrafish embryos utilizing CondD‐C involved 5 steps (**Figure** **6**A). First, high‐concentration CPA (10 nL of 80 wt% PG + 20 wt% MeOH) was microinjected into the yolk of a zebrafish embryo at the high cell stage, 3.3 h after fertilization,^[^ ^61^ ^]^ (0 min, Figure 6B) using robotic microinjection. In the previous protocol,^[^ ^58^, ^59^, ^62^ ^]^ this microinjection step included gold nanorods for laser rewarming of the embryos. The gold nanorods were not included since the focus of this demonstration was on vitrification. The custom‐built robotic microinjection system was a computer vision‐guided robot that used off‐the‐shelf components to fully automate the microinjection procedure.^[^ ^62^, ^63^, ^64^ ^]^ The CPA‐injected embryos (1 min, Figure 6B) were transferred into an incubator at 28 °C to allow for CPA diffusion inside the yolk. After a 3‐h recovery period, the embryos were placed on a cryomesh and immersed in a precooling bath (2.7 M PG + 1.2 M MeOH + 0.5 M trehalose) for 5 min. The embryo showed a dehydrated state (Figure 6B), effectively increasing the internal CPA concentration in the embryo. The zebrafish embryos and mesh were placed on Kimwipe to wick off excess CPA and vertically immersed in liquid nitrogen to vitrify (Figure 6A).

Vitrification of the zebrafish embryo was tested on stainless steel mesh and nylon mesh with a vertical plunge (see Experimental Section) (Figure 6C,D). A microscope with a camera was used to visualize the vitrified embryos.^[^ ^35^, ^65^ ^]^ The embryos in which ice formed appeared white in color, while the vitrified embryos were transparent (blue arrow, Figure 6C,D). The number of zebrafish embryos used for each individual test was *n* = 18–22, totaling ≈500. The vitrification rate was calculated based on visual assessment of microscope images. The stainless steel mesh achieved the highest vitrification rate at 64 ± 7%, which was statistically significantly higher than nylon mesh (29 ± 7%) (Figure 6E). The high vitrification rate achieved by stainless steel was due to the high cooling rate of conduction‐dominated heat transfer, which was higher than the CCR (Table 1 and Figure S5, Supporting Information). The experimental cooling rate of stainless steel was 2.9 ± 0.1 × 10^4^ °C min^−1^, which was 1.2 times higher than nylon mesh (1.3 ± 0.2 × 10^4^ °C min^−1^).

The increased cooling rate contributed to a higher vitrification rate. The variation in embryo size led to a few embryos not being vitrified on the CondD‐C due to differences in CPA diffusion and dehydration state. Two strikingly clear embryos on stainless steel (before vitrification) turned out to be entirely ice‐formed embryos (after vitrification, Figure 6C), possibly due to unsuccessful CPA diffusion in the yolk or CPA loading into the embryos. The copper mesh was used here to further check the importance of higher‐density mesh and thermal conductivity. Copper mesh with *D* = 50 µm, which produced a cooling rate of 2.9 ± 0.1 × 10^4^ °C min^−1^, was similar to stainless steel, but we observed a 20% lower vitrification rate (Figure S19, Supporting Information). This is due to the decreased contact area introduced by the larger pore size, shown as increased *R*_h_ (increased *A*_m_ in Equation 2), compared with mesh with a smaller wire diameter. This further highlights the importance of mesh geometry and supports that a higher‐density mesh (i.e., smaller pore size) is preferred to a reduced *R*_h_. Although the measured cooling rates were similar, we believe the difference in vitrification rates was due to the temperature uniformity within the biosystem, influenced by the contact area relative to each mesh pore. The higher *A*_m_ of the stainless steel mesh (increase with area ratio in Figure 2C) reduced the *R*_h_ and *R*_m_ compared with the copper mesh *D* = 50 µm, and so the temperature of biosystem during cooling was more uniform on stainless steel. Note that the nonuniform temperature cannot be directly measured because the diameter of the thermocouple tip (≈150‐200 µm) is larger than the mesh pore size. The vitrification rate of zebrafish embryos validated that the denser mesh can increase the cooling rate and vitrification rate of the biosystem, which is in line with our model predictions (Figure 2C). For the cryomesh design, pore size needs to be less than 50 µm based on the experiments, which can enhance the contact area and reduce *R*_m_ (Equation 3). However, the pore size should also be larger than 5 µm to ensure wicking of excess CPA, especially for a smaller wire diameter mesh. The pinning force generated by the mesh wire of a fixed area increases with the decrease in pore size, which reduces proper wicking of the CPA.^[^ ^40^, ^41^ ^]^ Moreover, the experimental cooling rate of the zebrafish embryo had a similar value as the cooling rate of the 1‐µL CPA droplet (Figure S19, Supporting Information). These measurements validated the use of a 1‐µL CPA droplet as a representative model system used in our physical characterization (Figure S19, Supporting Information). Meanwhile, based on the 20–30% vitrification rate achieved, a cooling rate of 1.3 × 10^4^ °C min^−1^ was estimated as the threshold cooling rate for zebrafish embryos loaded by this protocol (dashed line, Figure 6E), which was higher than the CCR of the CPA for zebrafish embryos (Table S5, Supporting Information).

As the thickness of the zebrafish embryo was more than 300 µm, conventional rewarming could not rewarm the embryos with these CPA loading conditions.^[^ ^59^ ^]^ For the loaded CPA, rewarming rates greater than 9.3 × 10^5^ °C min^−1^ were expected to avoid devitrification upon rewarming.^[^ ^65^ ^]^ Therefore, rewarming of the vitrified embryos was not attempted in this study. Nevertheless, the successful vitrification achieved by CondD‐C allows us to further investigate complementary rewarming technologies, such as cryomesh Joule heating^[^ ^35^ ^]^ or laser rewarming in a higher throughput configuration.^[^ ^60^ ^]^

### 2.8 Design and Physical Limits of the Cryomesh Platform Technique

We summarized the key results and design principles that describe the physical limits of conduction‐dominated cryomesh and enabled the successful cryopreservation of different biosystems (**Table** **2** and Table S7, Supporting Information, more details in Supplementary Information). To determine how to improve the cryopreservation protocol, we analyzed the achieved and potential viability of biosystems tested in this study. The high cooling rate of CondD‐C demonstrated the highest viability improvements in small biosystems (i.e., coral larvae, Figure S20A, Supporting Information), but also demonstrated advantages for the vitrification of *Drosophila* embryos (Figure S20B, Supporting Information) and zebrafish embryos (Figure S20C, Supporting Information). With the increase of biosystem size (above ≈200 µm), the CondD‐C has a good vitrification rate during cooling, but more rapid heating approaches^[^ ^35^, ^60^ ^]^ are required to further increase the viability during rewarming with low CPA concentration (below ≈20%).

**Table 2:** Summary of key results related to cryomesh design and performance.

Design factors	Considerations and tests	Reference
Thermal properties of cryomesh	Materials choice—thermal conductivity *k* ≥ 10 W m^−1^ K^−1^ (e.g., stainless steel, aluminum, diamond, or copper)	Figures 2 and 3
	Commercially available cryomesh material	Figure S8, Supporting Information
Physical dimensions	Wire diameter: *D* ≤ 50 µm	Figure 2
	Solid fraction of mesh: 0.65 ≥ *Ф* ≥ 0.5	Figure S7, Supporting Information
	Critical length scales	Table S2, Supporting Information
	Commercially available cryomesh sizes	Figure S8, Supporting Information
	Mesh physical properties	Table S3 and S8, Supporting Information
Achievable cooling rates	Mesh alone	Figure S13, Supporting Information
	Horizontal versus vertical plunge	Figures S11–S13, Supporting Information
	Biosystem thickness	Figures S9 and S13, Supporting Information
Achievable rewarming rates	Estimated rate (different materials and thicknesses)	Figure S15, Supporting Information
	Validation of estimated rate (s. steel and nylon)	Figure S16, Supporting Information
Scalability to larger cryomesh area	Impact of frame size	Figure S14, Supporting Information
Validation with model biosystems	Coral larvae (survival rate with threshold cooling rate)	Figure 4
	Drosophila embryo (hatch rate, vitrification rate with threshold cooling rate)	Figure 5 and Figure S17, Supporting Information
	Zebrafish embryo (vitrification rate with threshold cooling rate)	Figure 6 and Figure S19, Supporting Information
Further optimization	Mesh improvement opportunities	Table S4, Supporting Information
	Expected impact of improvements on biosystems	Figure S20, Supporting Information
	Physical limits of design	Figures S21 and S22, Supporting Information

In Figure S21A, Supporting Information, the dashed lines show the theoretical maximum cooling rate of different cooling methods. Between the conduction cooling and convection cooling regions is the CondD‐C cooling method reported in this study, which has a higher cooling rate than convection‐dominated cooling and fills the gap between the convection and conduction cooling methods (Figure S21A, Supporting Information). Increasing the thermal conductivity of cryomesh increases the cooling rate from the convection cooling region moving toward the conduction cooling region. With knowledge of the achievable cooling rate for different biosystems of different thicknesses, the CPA concentration can be further optimized (based on CCR and CWR), as shown in Table 1 and further illustrated in Figure S21B, Supporting Information. Note that a high CPA concentration increases the potential for toxicity in the biosystem while a low CPA concentration leads to the increased likelihood of devitrification with ice formation (Figure S21B, Supporting Information). By increasing the cooling rate of the cryomesh (i.e., CondD‐C), the lowest CPA concentration required for vitrification can be reduced, especially, for a smaller biosystem with a thickness <200 µm (blue dashed line, Figure S21B, Supporting Information). Thus, the yellow‐colored region between the red and blue dashed lines is defined as the cryomesh optimal zone. With cryomesh designed for general cryopreservation, the CPA concentration could be increased to facilitate successful vitrification based on the cryomesh optimal zone from Figure S22, Supporting Information, which reduces the CWR required. The concentration of the CPA loading determines the biosystem's limiting CCR and CWR (see Table 1). Knowledge of the limiting CCR and CWR can allow the selection of mesh designs to achieve these rates based on the characteristic size of the biosystem (Figure S21 and S22, Supporting Information).

As CWR is usually at least an order of magnitude higher than CCR, it is expected that cooling with the cryomesh will be achieved for some cases where warming cannot be achieved. Therefore, the limit of biosystem thickness can be determined based on a given CPA concentration, which is directly proportional to the CWR (Figure S22B, Supporting Information). As one example, assuming a CPA concentration equilibrated to 36 wt% (*Drosophila* final step CPA), the largest thickness of the biosystem (*L*_t_) that can be rewarmed without ice formation is around 400 µm based on theoretical calculation. As a general selection of biosystem thickness, the thickness should be less than 500 µm with a CPA concentration of 40 wt%. It also should be noted that different CPA cocktails will have different CWR, which can change the limit of the biosystem thickness. These examples provide a first‐order analysis that can be used to generally estimate design principles. Under these conditions, ancillary techniques such as Joule heating or laser rewarming can be used to increase the achievable rewarming rates (Figure S22, Supporting Information). The flowchart in Figure S23, Supporting Information demonstrates steps to modify the cryopreservation protocol for cryomesh to improve the viability of cryopreservation. The summary of the key results and design principles in Table 2 provides validation for these design considerations.

The critical cryomesh design parameters, including filament diameter, pore size, and material, can be optimized based on biosystem size. This can include, the biosystem size (e.g., the diameter of the biosystem or the minor axis) should be larger than the mesh pore size, which has a ratio (biosystem size/pore size) ≥2 with the largest pore size of 200 µm. The filament (wire) diameter should be smaller than the biosystem thickness, which has a ratio (diameter/thickness) ≤1 with the largest diameter of 50 µm. The mesh material should have a thermal conductivity of *k* ≥ 10 W m^−1^ K^−1^. For example, coral larvae have a diameter of ≈100 µm. Thus, we choose stainless steel mesh with a wire diameter of around 30 µm and a pore size of 35 µm. To improve the performance of the cryomesh, we considered additional parameters for design and modification, including hydrophobicity of the mesh, mechanical properties, adhesion rate, and wash‐off rate (Figure S24 and Table S8, Supporting Information). In this study, we mainly focused on investigating how to use the fundamental understanding of heat transfer to improve the viability of cryopreservation. Thus, as the next phase of the study, we will investigate 1) the biocompatibility of cryomesh more comprehensively with assessments such as cytotoxicity, proliferation, and potential immune responses and 2) a more comprehensive analysis of the long‐term effect and potential damage of cryopreservation.

## 3 Conclusion

We report a conduction‐dominated cryomesh technology and approach that achieves vitrification‐based cryopreservation for different organisms, including coral larvae, *Drosophila* embryos, and zebrafish embryos. The cooling rate is enhanced by the high thermal conductivity of cryomesh, the design of the cryomesh geometry to facilitate heat transfer from the biosystem, and the modified plunge technique, which mitigates the effects of the LN_2_ vapor barrier during cooling. Based on the 1D heat transfer model, we identify design principles for conduction‐dominated cryomesh, including: 1) high thermal conductivity cryomesh material (*k* ≥ 10 W m^−1^ K^−1^) to achieve conduction‐dominated behavior; 2) small wire diameter (*D* ≤ 50 µm) and solid fraction (*Ф* = 0.5) to increase the heat transfer area, ensure adequate contact with the biosystem, and reduce the thermal resistance of the cryomesh; and 3) vertical plunging method with enhanced bubble release to achieve higher convective heat transfer rates, which enhance the heat release of the biosystem into LN_2_. Thus, stainless steel with a wire diameter of 30 µm and solid fraction of 0.5 achieves a cooling rate of 3.5 × 10^4^ °C min^−1^ for a 1‐µL CPA droplet, which is 3.2× the cooling rate of the convection‐dominated cryomesh with the horizontal plunge. With the enhanced cooling rate and vertical plunge, uniform cooling for scaled‐up meshes (i.e., 15 × 4 cm) was achieved based on thermally conductive materials and mesh design. Meanwhile, these design principles were also applied to study rewarming rates, showing the potential for comparable increases over the convection‐dominated cryomesh. Unsuccessful CPA diffusion inside the biosystem shows a fluctuation of survival rate near the threshold cooling rate, which requires a higher CCR and CWR and can be reached by CondD‐C. By applying these concepts, the successful vitrification of coral larvae, *Drosophila* embryos, and zebrafish embryos at higher rates than previous protocols were demonstrated. For instance, for coral larvae post‐warming viability was increased from 43% to 85%, with the added benefit of a scalable platform to potentially cryopreserve large quantities in a single loading. The scale‐up achieved by conduction‐dominated cryomesh paves the way to cryopreserve a wide range of organisms in greater quantities. This work not only demonstrates the effectiveness of a conduction‐dominated cryomesh to enhance the cooling and rewarming rates but also provides a paradigm for cryopreservation designs from a thermal perspective.

## 4 Experimental Section

### Physical Fabrication of Cryomesh

Cryomesh was fabricated with a 3D‐printed frame with different sizes of mesh. Before fabrication, the metal mesh was cleaned with acid dip solution (Rio Grande, prepared according to manufacturer directions) for 1 min and cleaned with DI water for at least 2 min. The PLA frame was designed using Autodesk Fusion 360 and 3D printed on a LulzBot TAZ 6 3D printer. All meshes, metal, and nylon, were fused with a soldering iron to the frame at a temperature of ≈250 °C using a digital soldering station with a controlled temperature (Radioshack). The temperature of the iron was within the range of the glass transition temperature of PLA. By pressing the mesh, the softened PLA frame can penetrate through the mesh pores. Thus, the mesh was bonded firmly with the PLA frame. The cryomesh was further cleaned with 75% ethanol and DI water, successively. Copper mesh and stainless steel mesh were purchased from TWP Inc. Nylon mesh was purchased from McMaster–Carr.

### Cooling Rate Measurement

To measure the cooling rates of the cryomesh method, a bare wire type‐T thermocouple (unsheathed fine gauge thermocouple, 50 µm wire diameter, OMEGA), and an oscilloscope (DS1M12, USB‐Instruments) were used. Cooling and warming rates were calculated from −140 to −20 °C using Microsoft Excel. The cooling rates of the coral larvae could not be directly measured due to limited access to coral larvae. Meanwhile, the thermal property of dehydrated coral larvae was unknown. The thermal property of CPA is used based on previous studies,^[^ ^31^, ^66^ ^]^ which had a similar thermal conductivity value to PE (0.3–0.5 W m^−1^ K^−1^).^[^ ^67^ ^]^ To estimate cooling rates, PE (polyethylene) particles (Cospheric LLC) immersed in CPA were used to simulate the vitrification of coral larvae and measured the cooling rate, which had a similar diameter of coral larvae with a diameter of 100 µm.

### Cryopreservation Protocol for Coral Larvae

Production of coral larvae: 20 individuals of the Hawaiian solitary mushroom coral, *Lobactis scutaria*, obtained in accordance with Hawaii Department of Land & Natural Resources Special Activity Permit 2023–31, were placed in separate bowls at 16:00 local time 1 and 2 days after the full moon in August and September 2022, covering the known times of spawning for that species in Hawaii.^[^ ^4^ ^]^ 2 days after the full moon, between 17:00 and 18:30, the corals spawned by releasing brief puffs of eggs or sperm. Eggs from females were captured on release with a transfer pipette directly from the mouths of the corals and transferred to clean bowls with 0.5‐µm‐FSW. Sperm was collected from male bowls with a transfer pipette and pooled into a separate bowl. Pooled sperm were added into the egg bowls for a final egg‐to‐sperm ratio of ≈1:10000 and left to fertilize for 1 h. The fertilized embryos were gently rinsed to remove as much sperm as possible and were left to develop in a 26 °C environment. Daily cleaning with FSW maintained the larvae in good health.

### Mesh Vitrification of Coral Larvae

One of the CondD‐Cs tested was a copper mesh. It was noted that copper shows toxicity in marine organisms by affecting their metabolic processes.^[^ ^68^ ^]^ Thus, before vitrification, the toxicity of the mesh to coral larvae was tested (Figure 4B). An Olympus SZX10 stereo microscope and Science Supply S01‐0801A camera were used to visualize the coral larvae morphology, which can be used to determine toxicity (i.e., swimming or not).^[^ ^4^ ^]^ For this investigation, the coral larvae were placed on the copper mesh for less than 2 min, directly immersed in seawater, and then removed for assessment. After exposure to the copper mesh, the outer edge of the larvae appeared dissolved, with a poorly defined border (Figure 4B). The larvae showed no survival following exposure to the copper mesh. The nylon and stainless steel mesh did not demonstrate any toxicity under similar conditions.

Mesh cooling and warming were used to cryopreserve and return larvae of *L. scutaria* to physiological conditions. The vitrification solution was used successfully in a previous study to cryopreserve larvae of the same species by vitrification and laser warming:^[^ ^4^ ^]^ 10% v/v propylene glycol + 5% v/v dimethyl sulfoxide + 1 M trehalose prepared in phosphate‐buffered saline (vitrification solution, VS) and 0.5 M trehalose prepared in FSW (rewarming solution, RW). Preliminary trials were performed with nylon mesh with *D* = 50 µm and *Ф* = 0.5, stainless steel mesh with *D* = 50 µm and *Ф* = 0.33, and stainless steel mesh with *D* = 30 µm and *Ф* = 0.5 (two technical replicates of each mesh type) on larvae between 3 and 4 days of development. Larvae were moved on CondD‐C into VS for a 2‐min exposure, followed immediately by wicking of excess VS and vertical plunging into liquid nitrogen. Larvae were rewarmed by vertical plunging into RW, left in RW for 2 min, and returned to FSW to recover. For the preliminary mesh trials, 50–200 larvae were placed on each mesh, and the mesh was immersed in 0.22‐µm‐filtered seawater. The water level was such that the swimming larvae could not leave the mesh frame. The mesh was removed from the seawater and gently dabbed from underneath with a Kimwipe and a Q‐tip cotton swab to remove residual FSW. The mesh was immediately transferred into a 35 mm dish containing vitrification solution (VS: 10% v/v PG, 5% v/v DMSO, and 1 M trehalose in phosphate‐buffered saline) and left for 2 min. The mesh was removed from VS, dabbed again with a Kimwipe and a Q‐tip cotton swab, and immediately plunged vertically into liquid nitrogen. When the excess CPA is wicked away from the cryomesh, a small amount of viscous CPA remains and adheres the coral larvae to the cryomesh by surface tension.

### Warming of Coral Larvae and Survival Assessments

Cooled forceps were used to retrieve the mesh from the liquid nitrogen bath. The mesh was briefly (<1 s) shaken to remove residual liquid nitrogen and immediately plunged into a rehydration solution of 0.5 M trehalose in FSW and left for 2 min. The mesh was removed from the rehydration solution, dabbed from underneath with a Kimwipe and a Q‐tip cotton swab, transferred to FSW for larval recovery, and percent survival was assessed by eye at 2 h post‐thaw. When the coral larvae are rewarmed, the cryomesh with coral larvae are transferred to the unloading solution. The viscosity of the CPA is reduced and does not produce enough surface tension to hold the coral larvae. Thus, when performing the last unloading in fresh seawater, all coral larvae detach from the cryomesh. No larvae were observed to be remaining on the cryomesh after 2 h of post‐thaw. Survival was calculated as the number of larvae that demonstrated active swimming divided by the total number of larvae present in the field of view.

### Cryopreservation Protocol for Drosophila Embryos

A *Drosophila melanogaster* stock derived from the *w* ^1118^ strain called M2 was used in this study.^[^ ^31^, ^35^ ^]^ The protocol follows previously reported protocols.^[^ ^31^, ^35^ ^]^ The *Drosophila* embryos were collected on a grape juice plate for 1 h. The plate was incubated in a 20 °C incubator until the youngest embryos reached 22 h old. The embryos were dechorionated with 50% bleach (1:1 mixture of DI water and Clorox disinfecting bleach) for 3 min and rinsed with water. Before CPA loading, the embryos were permeabilized with isopropanol (ACS reagent ≥99.5%, Sigma), 1:4 v/v of d‐limonene (food grade, Blubonic Industries) and heptane (HPLC, Sigma), and heptane, successively. There were two CPA loading steps. The first CPA loading step involved incubation in 13 wt% EG prepared with cryobuffer^[^ ^31^ ^]^ at room temperature for 25 min. The embryos were transferred to the dehydration CPA (27 wt% EG + 9 wt% sorbitol in cryobuffer) on ice for 9 min. For all the above steps, the embryos were kept in a nylon mesh basket that was transferred between solutions so that the embryos were suspended in these solutions. Then, the CPA‐loaded dehydrated embryos were transferred to different cryomeshes (either nylon or stainless steel), and extra CPA was removed with a Kimwipe for further vitrification. The remaining CPA between embryos and cryomesh kept the embryos attached to the cryomesh in liquid nitrogen. A microscope (Amscope) and a CMOS C‐mount camera (MU1000) were used to visualize the vitrification of embryos on cryomesh set in liquid nitrogen.

### Cryopreservation Protocol for Zebrafish Embryos

Wild‐type zebrafish (*Danio rerio*) embryos were obtained from the University of Minnesota Zebrafish Core Facility. All animal care and welfare met NIH animal care standards. Full details of the approved protocols are listed with the Zebrafish Core IACUC (protocol # 1506–32642A). Previous protocols were used to establish cryopreservation procedures for zebrafish embryos and were modified for use with the cryomesh.^[^ ^58^, ^59^, ^65^ ^]^ Zebrafish embryos were microinjected with 10 nL of CPA at the high cell stage (80% PG and 20% MeOH) using robotic microinjection.^[^ ^69^ ^]^ The embryos were cultured in an incubator at 28 °C for 3 h after microinjection. The zebrafish embryos were transferred to the cryomesh with care. All embryos remained adhered to the cryomesh. The excess media was wicked away with a Kimwipe. Because high temperatures can reduce survival, wicking took no more than 20 s. The cryomesh was then immersed in a pre‐cooling bath containing 2.7 M PG, 1.2 M MeOH, and 0.5 M trehalose (Tre) with zebrafish embryo medium for 5 min.^[^ ^59^ ^]^ Following the pre‐cooling bath, a Kimwipe was used to wick out as much of the pre‐cooling bath solution as possible without injuring the embryos. The wicking process was completed in less than 20 s. The cryomesh was vertically plunged in LN_2_ with the CPA‐loaded and dehydrated zebrafish embryos attached. The vitrified embryos were cryopreserved at this stage and could be stored in liquid nitrogen for future use. To assess the vitrification rate, the cryomesh and zebrafish embryos were examined under a microscope while cryogenic temperatures were maintained in cold nitrogen vapor. The photos were captured using an overhead microscopic camera, thereby allowing measurement of the vitrification rate (Figure 6C,D). For each individual test, 20 zebrafish embryos were loaded on a single mesh. The maximum number of zebrafish embryos tested was *n* = 45 per 2 × 2 cm mesh. At least four independent tests were performed on ≈500 zebrafish embryos in total.

### Statistics

Experimental data were presented with mean values unless specified. For plots with two dependent variables, one‐way ANOVA (analysis of variance) and the Tukey test were used for statistical analysis using OriginLab. *P*‐values <0.05 were considered statistically significant.

## Conflict of Interest

Z.G., N.Z., M.L.E., and J.C.B. are inventors on a pending patent application (US Provisional Patent Application Serial No.: 63/431,241). S.B.K. and A.S.J. are founders of the company Objective Biotechnology. The authors declare that they have no other competing interests.

## Supporting information

Supporting Information

Supporting Information

## Data Availability

The data that support the findings of this study are available in the supplementary material of this article.
