# Supplementary material for: Conduction‐Dominated Cryomesh for Organism Vitrification
Source: Adv Sci (Weinh). 2023 Nov 28;11(3):2303317. doi: 10.1002/advs.202303317 (PMC10797434; doi:10.1002/advs.202303317)
Supplement: Supplementary file 1 — Supporting Information [file ADVS-11-2303317-s001.pdf]

ADVANCED SCIENCE
Open Access

Supporting Information

for *Adv. Sci.*, DOI 10.1002/advs.202303317

Conduction-Dominated Cryomesh for Organism Vitrification

*Zongqi Guo, Nikolas Zuchowicz, Jessica Bouwmeester, Amey S. Joshi, Amanda L. Neisch, Kieran Smith, Jonathan Daly, Michael L. Etheridge, Erik B. Finger, Suhasa B. Kodandaramaiah, Thomas S. Hays, Mary Hagedorn and John C. Bischof**

Supporting Information

Table S1. Comparison of representative methods for cryopreservation of submillimeter biosystems.

Methods	Biosystem type	Cooling rate (°C/min)	CPA	Throughput	Ref
**Slow freezing**	Stem cell	1	10% DMSO	1 mL solution	[1]
	T-cell	1–10	CryoStor10	1 mL solution	[2]
	Oocytes/embryos	0.3–50	1.5 M	0.5 mL solution	[3]
**Cryotop**	Human embryos	2.3×10^4	VT601	0.1 µL droplet	[4]
**Straw**	Sperm	180	R18S3	10 µL/straw	[5]
**Droplet vitrification**	Fibroblast/myoblast cells	2.2×10^6	CPA free	4.8 µL/min	[6]
	Fibroblast cells	1.1×10^3	1.4 M DMSO	N/A	[7]
	Blood stem cells	1.75×10^4	2 M PG + 1 M trehalose	0.6 mL/min	[8]
**Cryomesh**	Islets	5.4×10^4	22% EG + 22% DMSO	4250 /cm^2	[9]
	Drosophila embryo	5×10^4	39% EG + 9% sorbitol	400 /cm^2	[10]

We quantified the cooling rates of direct-printing of droplets in LN_2, convection-dominated cryomesh (ConvD-C), and conduction-dominated cryomesh (CondD-C). A 1-µL CPA droplet was pipetted on the mesh and plunged into LN_2. The CPA concentration used was 14 wt % EG + 14 wt % DMSO + RPMI (Roswell Park Memorial Institute 1640 Medium). CondD-C shows the highest cooling rate at 2.4×10^4 °C/min, which is 144% higher than the convection-dominated cryomesh. The convection droplet shows the lowest cooling rate, which is 13% of the conduction-dominated cryomesh. Note, the cooling rate of the convection droplet is calculated based on the levitation time of the CPA droplet (the time the vapor barrier kept the droplet suspended in LN_2), as the cooling rate cannot be directly measured by thermocouples for this case. With the convective mesh and vertical plunge, the heat transfer behavior of the biosystem on the cryomesh is similar to the pure conduction heat transfer of a droplet printed directly on a pre-cooled plate.

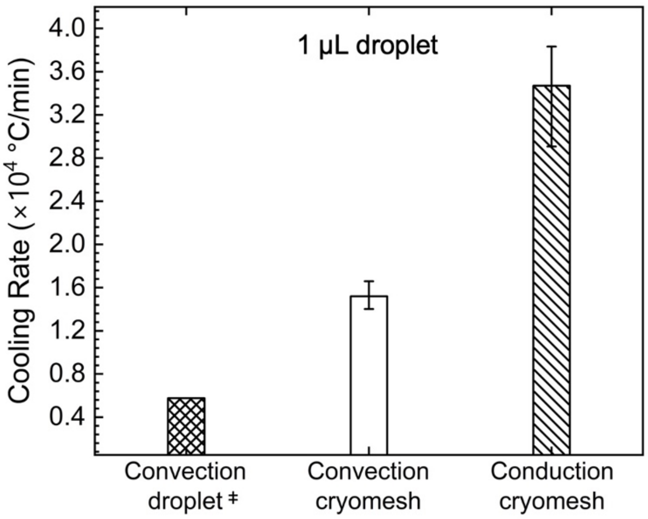

Figure S1. Experimental cooling rates of convection droplet, convection-dominated cryomesh, and conduction-dominated cryomesh. ǂNote: cooling rate of the convection droplet is calculated based on experimental levitation time. The error bar shows the range of the data. CPA used to show the difference between those methods has a concentration of 14 wt % EG + 14 wt % DMSO + RPMI (Roswell Park Memorial Institute 1640 Medium), which has been used previously in convective cryomesh experiments[9].

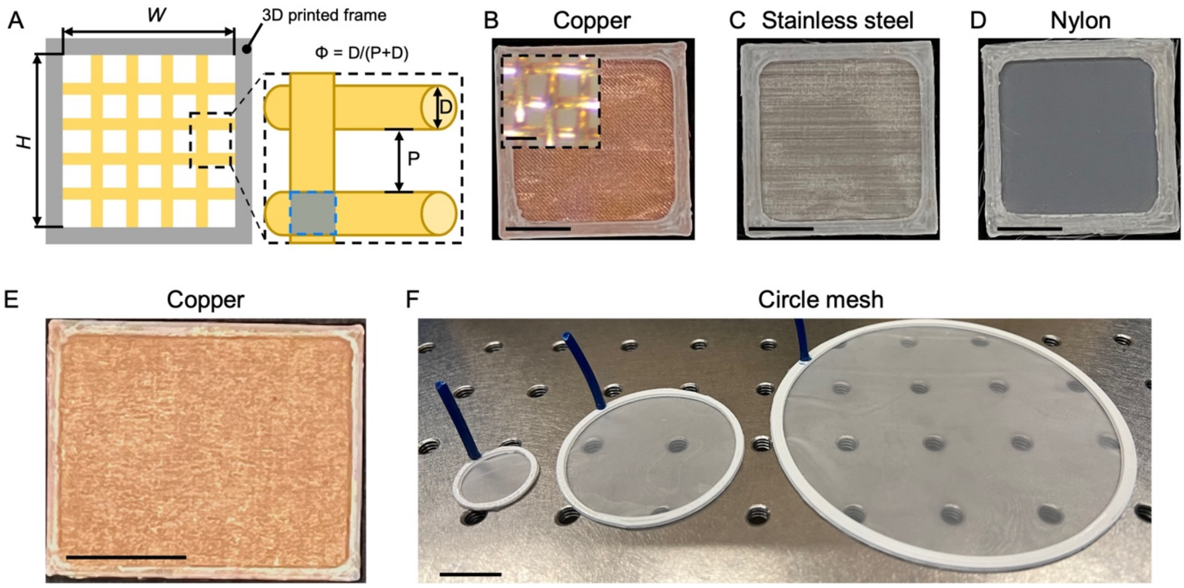

Figure S2. Description and examples of cryomesh used in this work. (A) Schematic of cryomesh showing definition of cryomesh height (*H*), width (*W*), filament diameter (*D*), pore size (*P*), and solid fraction (*Φ*). Camera images of (B) copper (2 × 2 cm), (C) stainless steel (2 × 2 cm), (D) nylon (2 × 2 cm), (E) copper (5 × 4 cm), and (F) various circular mesh used in this study. Scale bars are 1 mm for (B), (C), and (D) and 2 mm for (E) and (F). The scale bar of the insert in (B) is 100 µm.

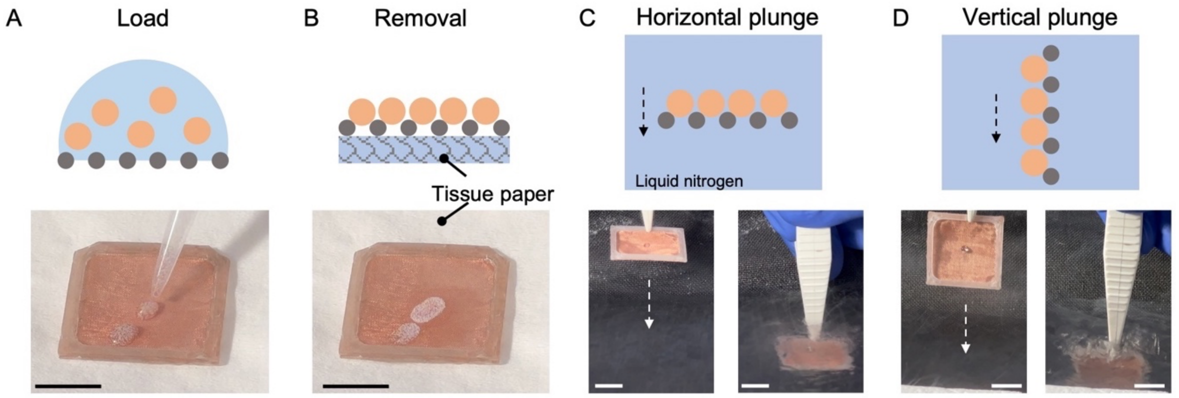

Figure S3. General vitrification steps for the cryomesh. An example is shown using 125 μm PE (Polyethylene) microspheres to model coral larvae. (A) The biosystem is loaded onto the cryomesh, which is shown here through direct pipetting but can also be loaded in suspension. (B) Excess CPA is removed by wicking with tissue paper (e.g., Kimwipe). (C) Horizontal plunging into liquid nitrogen is accomplished by plunging the cryomesh with the mesh plane parallel to the top surface of the liquid nitrogen bath. (D) Vertical plunging into liquid nitrogen is accomplished by plunging the cryomesh with the mesh plane perpendicular to the top surface of the liquid nitrogen bath. Scale bars are 1 mm.

Transient heat conduction analysis:

During cryomesh cooling in LN_2, there are two processes of heat release: 1) convection heat transfer, which releases the heat of the mesh to liquid nitrogen, and 2) conduction heat transfer inside and between the mesh and biosystem. We further studied the heat release of conduction-dominated cryomesh in liquid nitrogen with a 1D transient heat transfer model (analytical representation of Figure 2A). Thus, the traditional governing heat transfer equation is

$$\frac{\partial T}{\partial t} = \alpha \frac{\partial^2 T}{\partial x^2} \quad \text{S1}$$

with boundary conditions

$$\frac{\partial T(0,t)}{\partial x} = 0 \quad \text{S2}$$

$$-k\frac{\partial T(L_s,t)}{\partial x} = h[T(L_s,t) - T_\infty] \quad \text{S3}$$

and initial condition

$$T(x,0) = T_i = 0\sim 20\ ^\circ\mathrm{C}, T_\infty = -196\ ^\circ\mathrm{C} \quad \text{S4}$$

where T is the temperature; t is the time; α is the thermal diffusivity; k is the thermal conductivity; L_s is the thickness of the biosystem; and h is the convection heat transfer

coefficient. Note we assume the convection heat transfer is low on the other side of the biosystem and neglect it in this analysis. Based on the transient heat transfer Equation S1, we further simplified the heat transfer model to calculate the heat release time. The heat release time is defined here as the time for the whole system (mesh with biosystem) to cool down to the desired temperature (e.g., LN_2 temperature). We divided the total heat release time (t_{total}) into three parts:

$$t_{\mathrm{total}} = t_{\mathrm{c}} + t_{\mathrm{m}} + t_{\mathrm{b}} \qquad \text{S5}$$

where t_c is the heat release time from mesh to LN_2 through convection heat transfer, t_m is the heat release time for conduction through the mesh, and t_b is the heat release time for conduction through the biosystem. We calculated the heat release times similar to calculating a time constant (τ)[11], which is

$$\tau = \frac{\rho c_{\mathrm{p}} V}{hA} \qquad \text{S6}$$

where ρ is the density, c_p is specific heat, and V is the body volume. We assumed that all heat from the system (mesh with biosystem) is released from the mesh to LN_2 through convection heat transfer. We calculated t_c as

$$t_{\mathrm{c}} = \frac{Q_{\mathrm{m}} + Q_{\mathrm{b}}}{hA_{\mathrm{m}}} \qquad \text{S7}$$

where h is the convection heat transfer coefficient with a range from 250 to 2500 $W/m^2/K$[12]. The heat of the mesh Q_m is

$$Q_{\mathrm{m}} = \rho_{\mathrm{m}} C_{\mathrm{p,m}} V_{\mathrm{m}} \Delta T \qquad \text{S8}$$

$$V_{\mathrm{m}} = 4\pi \left(\frac{D}{2}\right)^2 L_{\mathrm{u}} \qquad \text{S9}$$

Where D is the mesh wire diameter and L_u is the unit length (Figure 2A). The heat of the biosystem Q_b is

$$Q_{\mathrm{b}} = \rho_{\mathrm{b}} C_{\mathrm{p,b}} V_{\mathrm{b}} \Delta T \qquad \text{S10}$$

$$V_{\mathrm{b}} = L_{\mathrm{s}} \cdot {L_{\mathrm{u}}}^2 \qquad \text{S11}$$

where L_s is the thickness of biosystem (Figure 2A). A_m is the contact area between the mesh and LN_2, calculated as

$$A_{\mathrm{m}} = (4\pi D L_{\mathrm{u}} - 4\pi D^2)/2 \qquad \text{S12}$$

The contact area considers one side of the surface area of the entire mesh and excludes the intersection area between each wire (shadowed area, Figure S2A). We calculated t_m as

$$t_{\mathrm{m}} = \frac{Q_{\mathrm{m}} \cdot D}{k_{\mathrm{m}} A_{\mathrm{cm}}} \qquad \text{S13}$$

where k_m is the thermal conductivity of the mesh and the cross-section area of mesh was calculated as

$$A_{cm} = \Phi \cdot {L_u}^2 \quad S14$$

where Φ is the solid fraction. The t_b was then calculated as

$$t_b = \frac{Q_b \cdot L_s}{k_b A_b} \quad S15$$

where k_m is the thermal conductivity of the mesh and A_b is the cross-section area as ${L_u}^2$. We assume CPA thermal properties are similar to water and the interfacial thermal resistance is considered between water and bulk material. Thus, the interfacial thermal resistance between cryomesh and CPA is ignored due to its small value ($4 - 8 \times 10^{-9}$ $m^2K/W << R_m$, 1 – 1000 m^2K/W)[13]. The cooling rate was calculated as

$$CR = \frac{\Delta T}{t_{total}} \cdot 60\ (°C/min) \quad S16$$

where ΔT is the temperature zone from −20 °C to −140 °C, which is generally the most relevant zone for vitrification and effective rewarming[8].

To further simplify the mesh definition, we could use critical length (L_C) to compare different mesh geometries. Based on Equations S9 and S12, we calculated the critical length as:

$$L_C = \frac{V_m}{A_m} \quad S17$$

Table S2 shows the L_C of different mesh filament diameters and solid fractions used in this study. Table S3 shows the thermal properties used for the calculation.

To further consider the convection heat transfer for the biosystem exposed directly to LN_2 (the side away from mesh), we divided the heat flow of the biosystem into 2 domains with heat flowing: 1) from biosystem to cryomesh and to LN_2 with the thickness of L_s (red rectangular, Figure S4A) named conduction-dominated region and 2) from biosystem directly into LN_2 showing a pure convection heat transfer with the thickness of $L_{s,c}$ (black rectangular, Figure S4A) named convection-dominated region. The lengths of these two regions were set by assuming the same heat release time with different biosystem thicknesses (e.g., $L_s \neq L_{s,c}$), which had a boundary condition ($\partial T(0,t)/\partial x = 0$) at $x = 0$ (i.e., the transition point between the two regions). We assumed the h was the same as on the mesh side. Note the experimental h for the biosystem might be lower than the assumption due to the nitrogen vapor layer trapped by the biosystem. Thus, we can assume the time for heat release time $t_{b,c}$ of biosystem through convection is

$$t_{b,c} = \frac{Q_{b,c} \cdot L_{s,c}}{k_b A_b} + \frac{Q_{b,c}}{h A_b} \quad S18$$

where $Q_{b,c}$ is the total heat of biosystem released by convection from biosystem side and $L_{s,c}$ is the length of biosystem affected by convection heat transfer. Based on the boundary condition (Equation S2), assuming $t_{total} = t_{b,c}$, we calculated the biosystem thickness affected by convection heat transfer, which is

$$L_t = L_s + L_{s,c} \quad \text{S19}$$

For the known biosystem thickness, e.g., the thickness of coral larvae was around 100 μm, we assigned L_t (total biosystem thickness) as the biosystem thickness of 100 μm. Then we combined Equations S5 and S18 to solve the cooling rate, where $t_{total} = t_{b,c}$. By assuming adiabatic in the first case (Figure 2A), we explore a worst case (i.e., slowest cooling rate), where convection is non-existent between biosystem and LN_2. While this case may be approached for some conductive meshes (i.e., resistance through cryomesh $R_m << R_{h,c}$ resistance between biosystem and LN_2), this idealization will always represent the slowest possible response. Nevertheless, this first case approximation allows easy correlation cooling response to mesh parameters (e.g., thermal conductivity, pore size, mesh diameter, etc.), to best articulate a cryomesh design principle.

To consider a more realistic case and validate the experimental results, we included the convection heat transfer from the biosystem to liquid nitrogen by assuming h was the same as on the mesh side (Figure S4A). In reality, we expect the nitrogen vapor bubbles to release more rapidly from the cryomesh and so this analysis might overestimate the effects of direct heat transfer from the biosystem to LN_2. However, since the heat transfer is dominated by conduction through the mesh, the predicted results are comparable to the experimental results and support the validity of our modeling approach. As one example, we used the model with adiabatic boundary condition (limiting case) to predict the cooling rate of a 1 μl droplet showing a reasonable agreement (Figure 3G).

Table S2. Critical length (L_C) of different mesh filament diameters and solid fractions.

Mesh size (μm)	Pore size (μm)	Solid fraction	Critical length (μm)	Materials
**30**	38	0.44	19	Stainless steel
**50**	50	0.5	33	Copper/Stainless steel/Nylon
**50**	100	0.33	30	Copper/Stainless steel/Nylon
**100**	200	0.33	67	Nylon

Table S3. Thermal properties[14, 15].

Material	Thermal conductivity (W/(m•K)	Density (kg/m^3)	Specific heat (J/(kg·K))	Thermal diffusivity (mm^2/s)
Diamond	2300	3510	520	1260.14
Copper	398	8954	380	116.97
Stainless Steel	16.3	8027	502	4.05
Nylon	0.2	1140	1670	0.11
CPA*	1.6	1100	1400	1.05
PE sphere	0.44	960	1330	0.34

*Due to the lack of data, the thermal properties are assumed to be a constant value for different CPA components/mixtures[16, 17]. We do not expect CPA thermal properties to vary significantly relative to other materials being compared here, so this should be a reasonable assumption. The thermal diffusivity (α) which is comprised of these properties was also constant and independent of temperature.

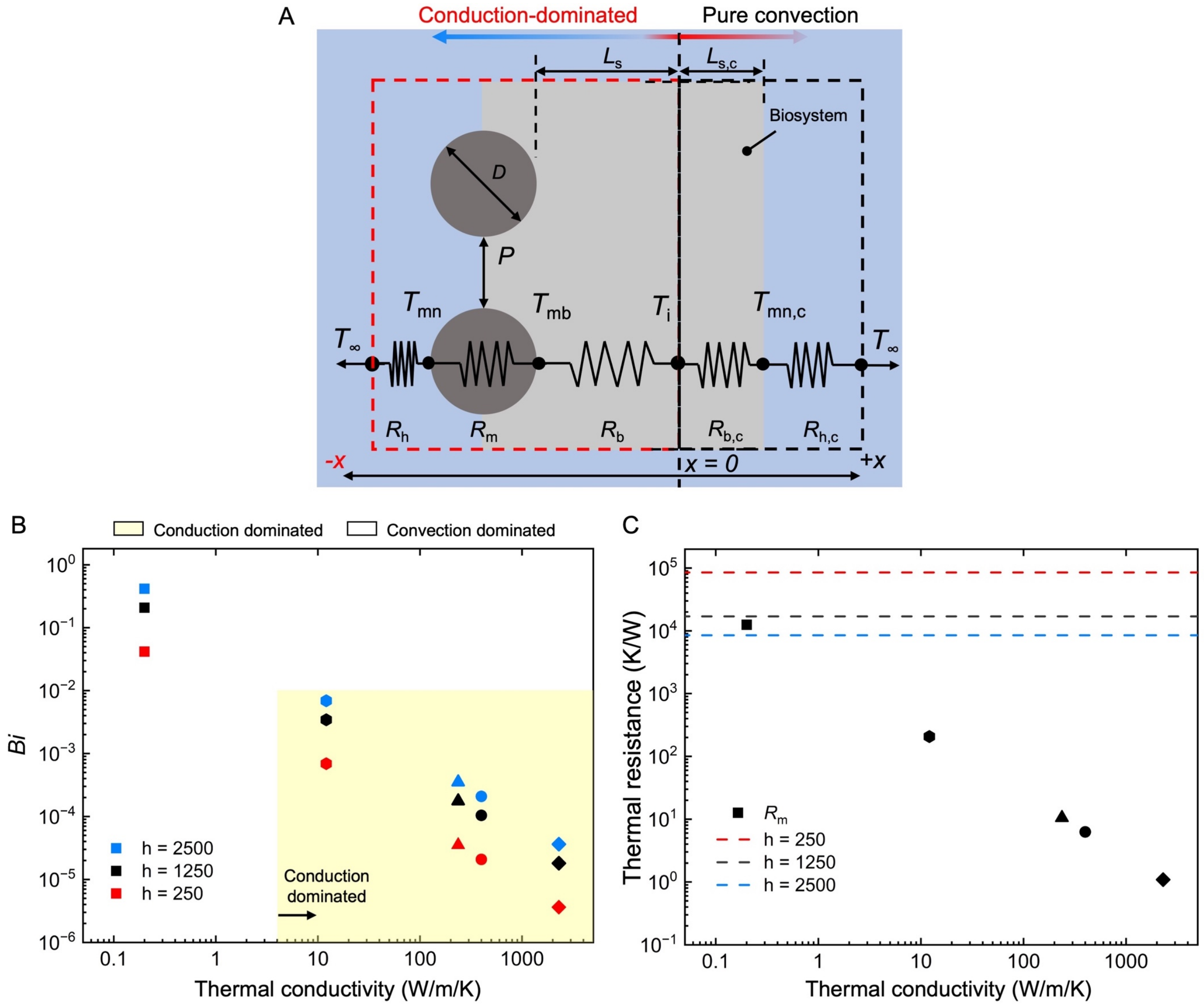

Figure S4. Modeling of heat transfer parameters for cryomesh with different thermal conductivities. (A) Heat transfer model of a cryomesh cooling in LN_2. L_s represents the biosystem thickness with cryomesh-enhanced conduction, while $L_{s,c}$ represents the biosystem thickness experiencing convection with LN_2. (B) Biot number calculated under different heat transfer coefficients for a range of thermal conductivities, assuming a characteristic length of 33 µm for mesh with D = 50 µm and Φ = 0.5. The yellow-colored area indicates conduction-dominated behavior, which has $Bi < 0.01$ and $h > 1250$ W/m^2/K. (C) Thermal resistances (K/W) of different heat transfer coefficients for a range of thermal conductivities, assuming a characteristic length of 33 µm. The x-axis is the thermal conductivity for different materials (W/m/K).

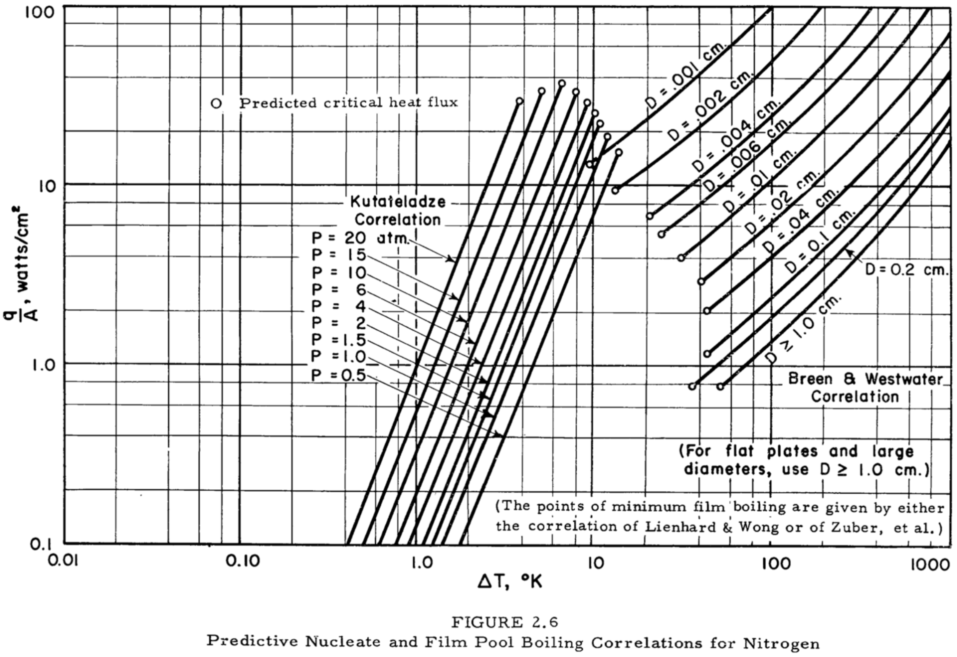

Figure S5. Predictive nucleate and film pool boiling curve of nitrogen. Adapted from Brentari et. al[18].

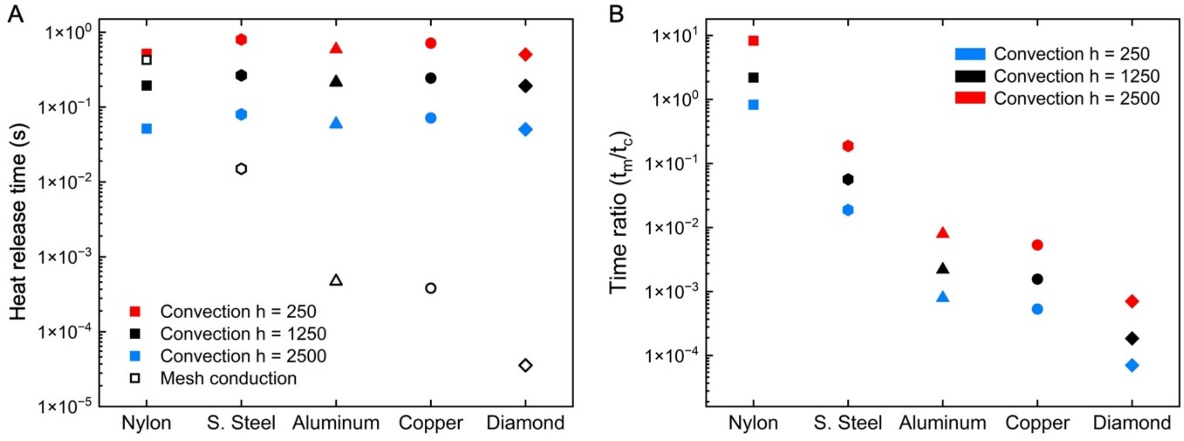

Figure S6. Modeling of heat release time. (A) Heat release time of 3 heat transfer coefficients for different materials. The heat release time is calculated from Equations S5–S15. (B) The ratio of conduction heat release time (Equation S13) and convection heat release time (Equation S7) for different materials correlate to the *Biot* number. The mesh has D = 50 μm and Φ = 0.5. Biosystem thickness is assumed to be 50 μm.

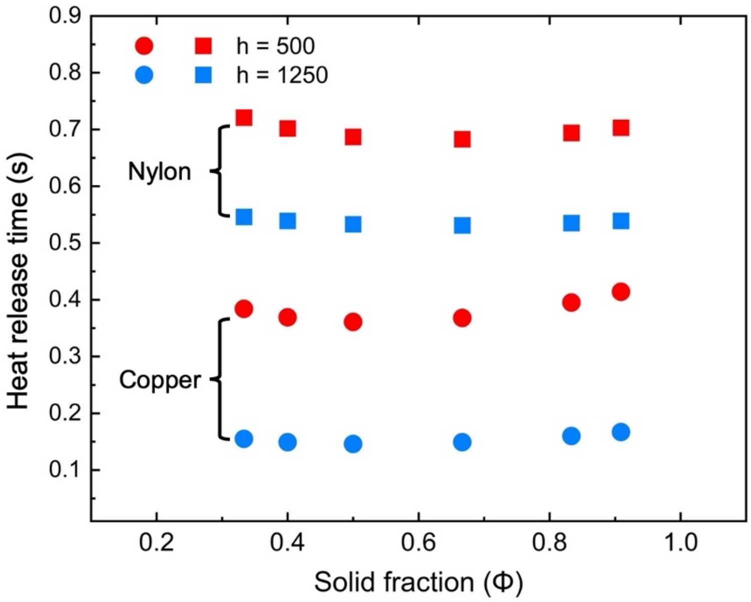

Figure S7. Heat release time (Equation S5) of copper and nylon mesh shown for a range of cryomesh solid fractions. The biosystem thickness is 50 µm. See Figure S2 for the definition of cryomesh solid fraction Φ. The wire diameter here is D = 50 µm.

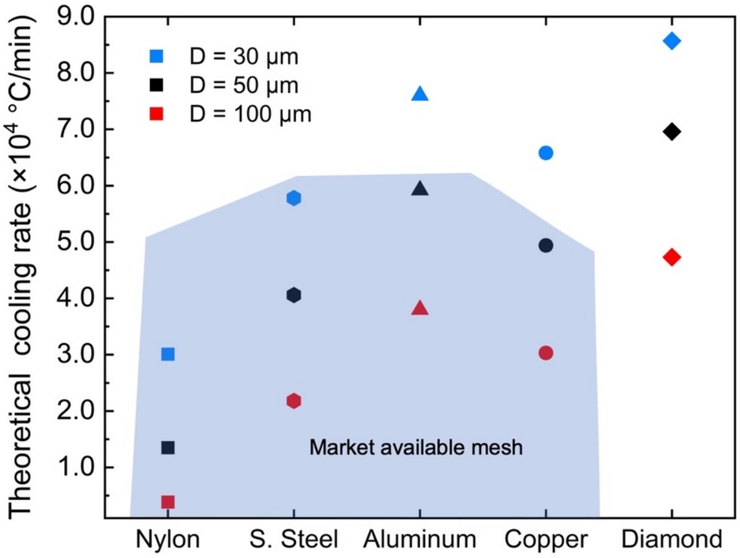

Figure S8. Theoretical cooling rate of different cryomesh materials with different mesh diameters. The cooling rate is calculated from Equation S16 with 50 µm biosystem thickness, where Φ = 0.5 and h = 1250 W/m^2/K. The colored area shows commercially available meshes. While experimental demonstrations focused on the comparison of nylon, stainless steel, and copper, aluminum offers another viable option for the conduction-dominated cryomesh. We decided that nylon, stainless steel, and copper provided an adequate range of behaviors; however, aluminum will be considered in future cryomesh designs.

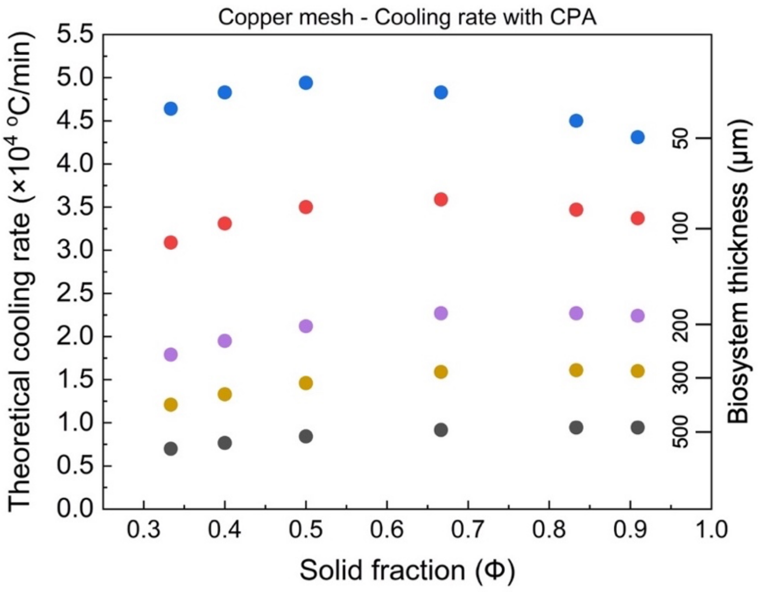

Figure S9. Theoretical cooling rate of different biosystem thicknesses with different solid fractions on a copper mesh with D = 50 μm and h = 1250 W/m^2/K. The theoretical cooling rate of different biosystem thicknesses is reported as the minimum cooling rate experienced. Data for different biosystem thicknesses corresponded to blue (50 μm), red (100 μm), purple (200 μm), gold (300 μm), and black (500 μm) dots.

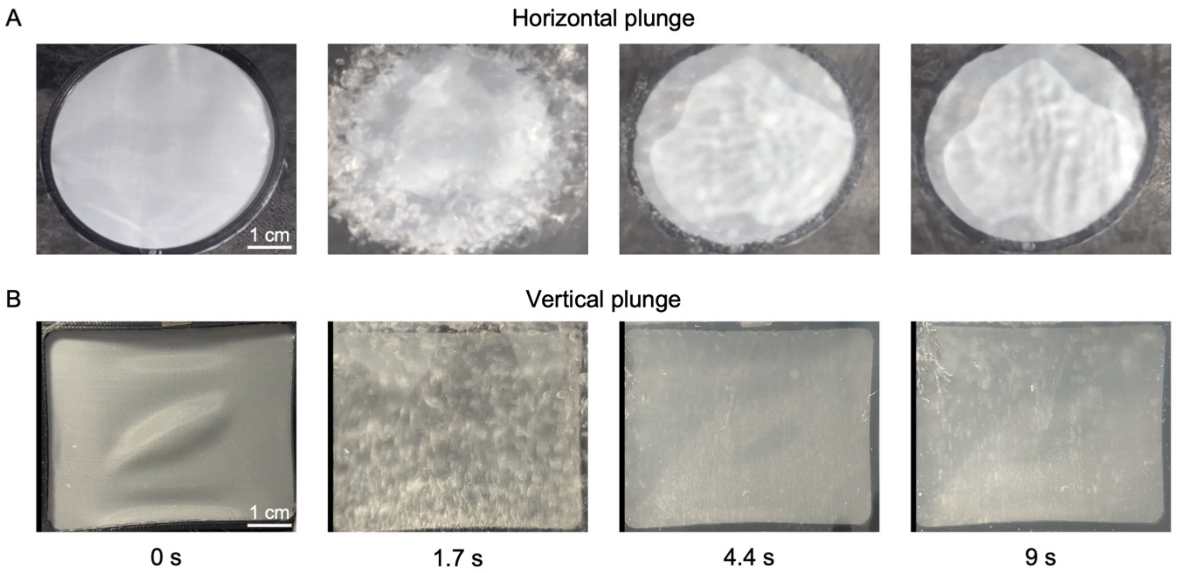

Figure S10. Horizontal plunge (A) and vertical plunge (B) into liquid nitrogen. The mesh is nylon mesh with D = 50 μm and Φ = 0.5. Vapor nitrogen is trapped by mesh during horizontal plunge, while all nitrogen bubbles release from the mesh during vertical plunge.

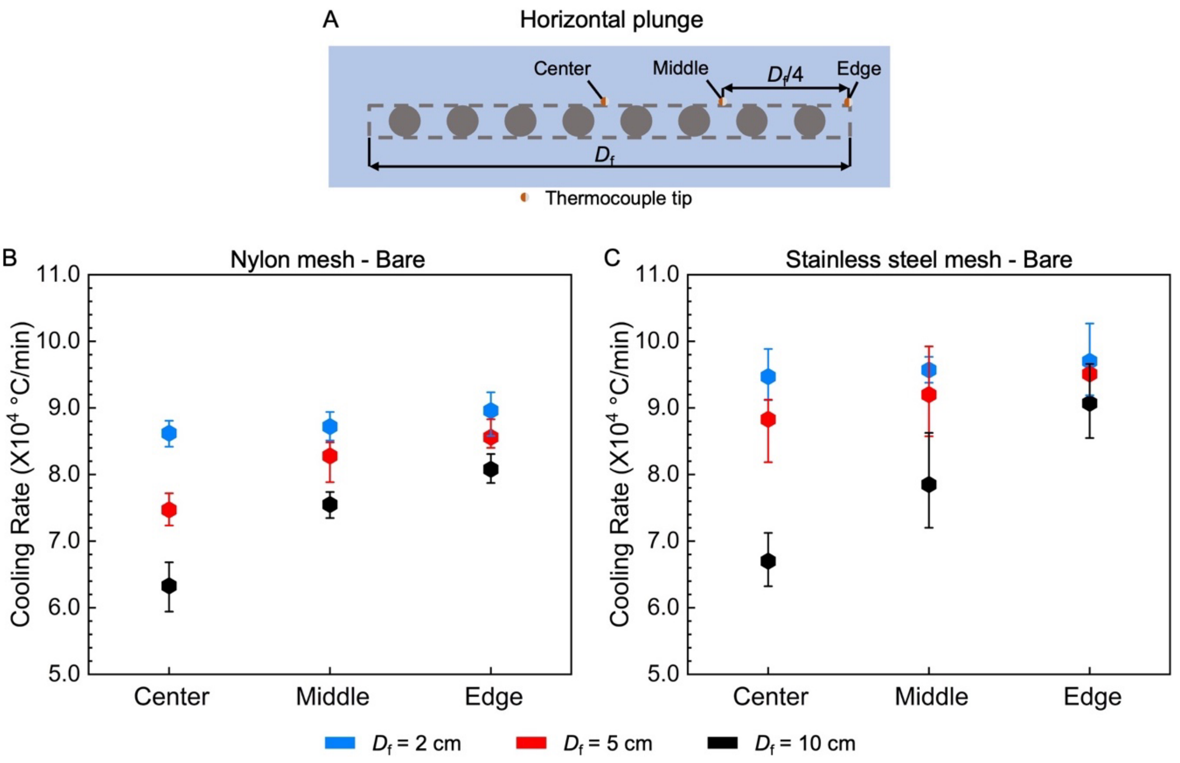

Figure S11. Temperature variation measured across various sizes of cryomesh during horizontal plunging of bare mesh without CPA loading. (A) Schematic of temperatures measured at three uniformly distributed points across the circular mesh center, middle, and edge. D_f is the diameter of the cryomesh frame. The thermocouple tip was attached to the mesh at the relative locations shown. (B) Plot showing cooling rate across the nylon cryomesh (D = 50 μm and Φ = 0.5) at three points from center to edge. (C) Plot showing cooling rate across the stainless steel cryomesh (D = 50 μm and Φ = 0.5) at three points from center to edge. The error bar is the range of the data.

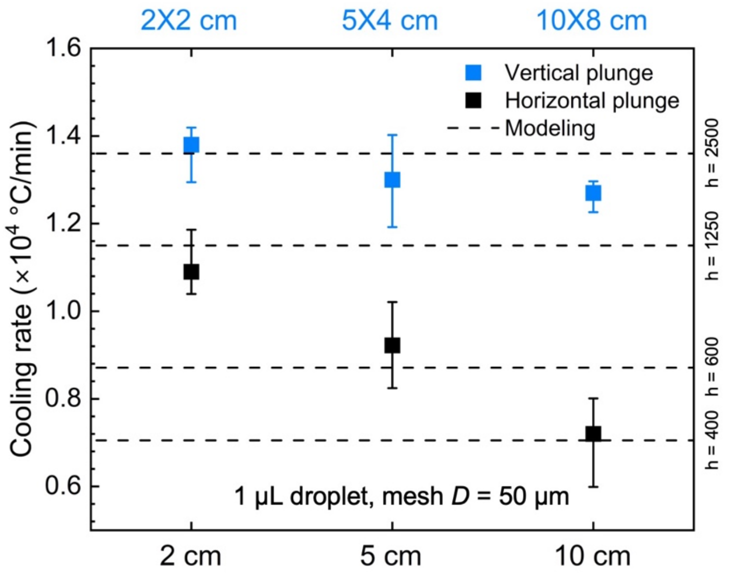

Figure S12. Measured cooling rates for vertical and horizontal plunge varying with mesh frame sizes. A 1-μL CPA droplet (14 wt % EG + 14 wt % DMSO + RPMI) was pipetted on the nylon mesh to simulate the largest biosystem tested. For horizontal plunge (black squares), the mesh frames were a circle shape with diameters from 2 to 10 cm. The model suggests a lower effective heat transfer coefficient on the horizontal plunge due to trapped bubbles.

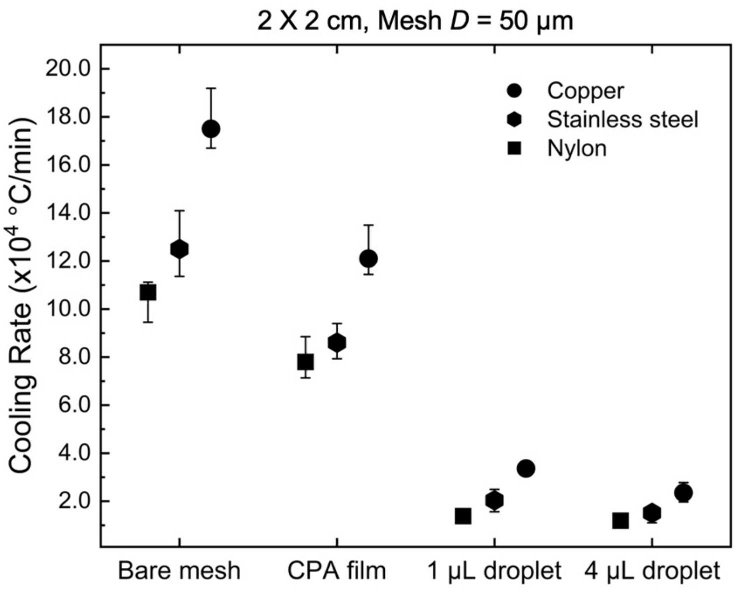

Figure S13. Measured cooling rate for the vertical plunge with different cryomesh materials of nylon, stainless steel, and copper. The cryomesh had a wire diameter of 50 μm and $\Phi = 0.5$. The cryomesh frame size was 2 × 2 cm. CPA film was loaded on the mesh by immersing the mesh into CPA solution and removing extra CPA with a Kimwipe. The CPA film coated the mesh with a thickness of ~ 2 μm, and a 1- or 4-μL CPA droplet (14 wt % EG + 14 wt % DMSO + RPMI) was pipetted on the mesh. The cooling rates of different biosystem sizes show a similar trend. The error bar is the range of the data.

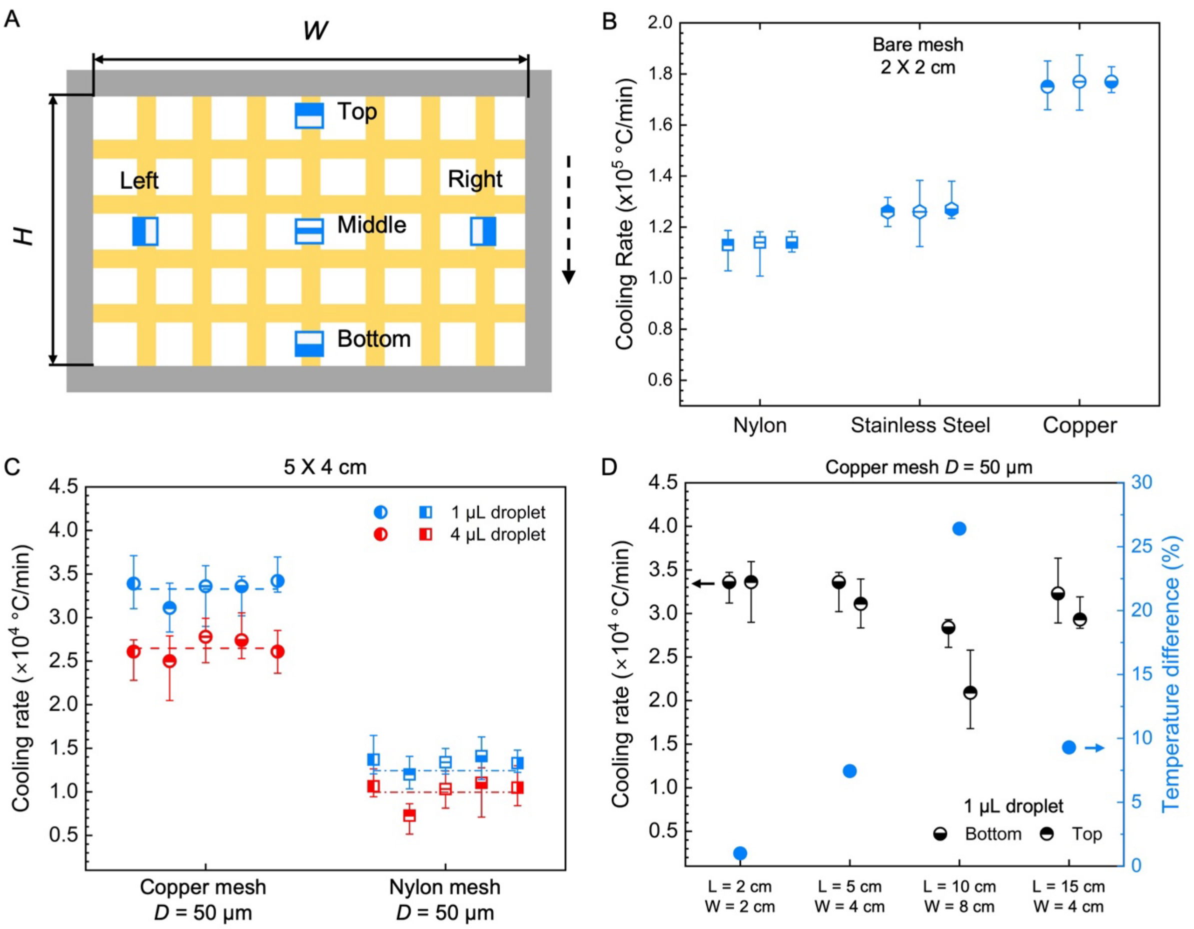

Figure S14. Cooling rate measured across different frame sizes of cryomesh during vertical plunging. (A) Schematic of the cooling rate measured at five uniformly distributed points across the mesh area. *L* is the length and *W* is the width of the frame. The arrow shows the plunge direction. The cooling rate is measured from −20 °C to −140 °C. (B) Cooling rate of the different bare meshes at three uniformly distributed points across the mesh at the top, middle, and bottom. The frame size is 2 × 2 cm. The wire diameter is 50 μm and $\Phi = 0.5$. The vertical plunge demonstrated uniformity across the mesh area. (C) Cooling rate measured across 5 × 4 cm (*L* × *W*) cryomesh during vertical plunging with a 1- or 4-μL CPA droplet. The horizontal dashed line shows the average cooling rate of the measured five points. The conduction-dominated cryomesh (copper) demonstrated uniformity across the area within 6% (difference between highest and lowest value), while the convection-dominated cryomesh (nylon) demonstrated non-uniformity with variation up to 34%. (D) Cooling rate measured across different frame sizes of conduction-dominated cryomesh during vertical plunging with a 1-μL CPA droplet. The wire diameter is 50 μm and $\Phi = 0.5$. To scale up the mesh, a short frame size (W ≤ 4 cm) is desired, as it shows a small temperature difference across the mesh (≤ 10%).

Table S4. Possible further improvements for cryomesh designs.

Problems	Improvement	Mechanism	Reference
Biosystem not vitrified	Increase convection heat transfer coefficient	Reduce bubble adhesion	[19-22]
		Wicking provides continuous liquid film	[23-28]
		Higher temperature difference	[10, 18]
	Increase cryomesh heat transfer	Reduce cryomesh thermal resistance	*
		Increase contact area for heat release	[25], *
Ice formation during rewarming	Rapid rewarming	Rapid rewarming of substrate	[17]
		Volumetric rewarming	[29]

*This work.

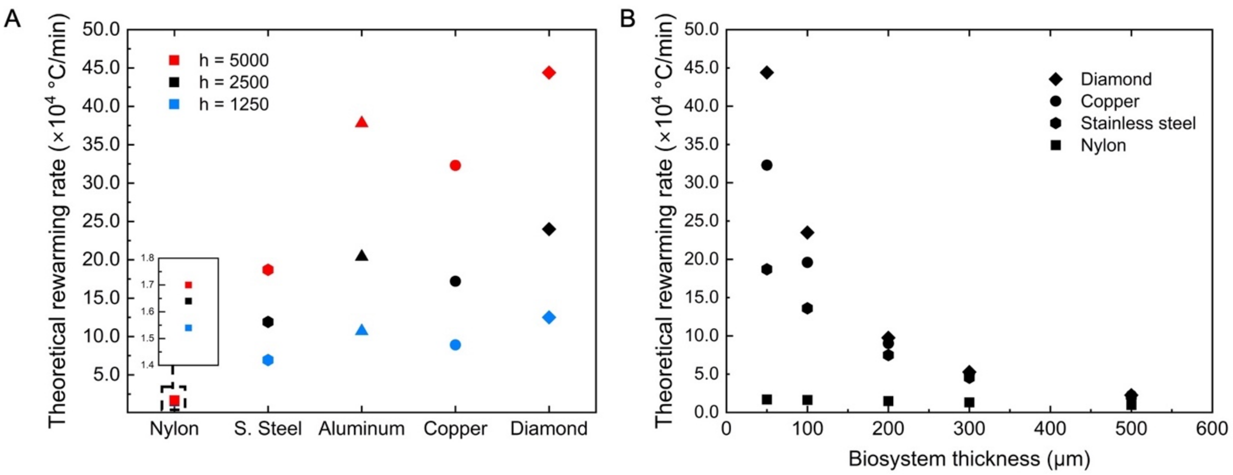

Figure S15. Theoretical rewarming rate estimated for a range of conditions. (A) Theoretical rewarming rate for a range of heat transfer coefficients for different materials, where D = 50 μm and Φ = 0.5. Rewarming rate shown is for the biosystem with a 50 μm thickness. (B) Theoretical rewarming rate for a range of biosystem thicknesses for different materials, where h = 5000 W/m^2/K, D = 50 μm, and Φ = 0.5. The simulated biosystem rewarming rate is reported as the minimum cooling rate experienced by the biosystem.

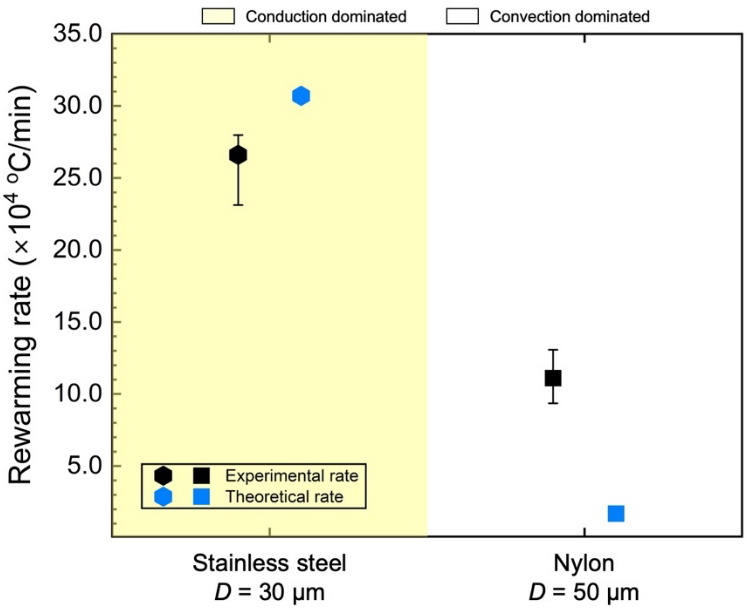

Figure S16. Validation of theoretical and experimental rewarming rate on stainless steel with *D* = 30 μm and nylon mesh with *D* = 50 μm. The experimental data is measured based on PE (polyethylene) particles. The thickness used in the model is 50 μm to consider the rewarming from the other side, which is different from the cooling model. We assume *h* = 5000 W/m^2/K to consider the conduction and convection boundary conditions when plunging into rewarming solution.

As this study mainly focuses on vitrification and cooling rate enhancement, we only validated the rewarming rate on stainless steel *D* = 30 μm and nylon mesh *D* = 50 μm as representative of CondD-C and ConvD-C, respectively (Figure S16). For CondD-C (stainless steel), the theoretical rewarming rate shows good agreement with the experimental rewarming rate. The experimental rewarming rate of ConvD-C (nylon) is higher than the predicted rewarming rate. Since the direct convective transfer between the biosystem and rewarming fluid was neglected, as discussed earlier, the predicted rewarming is lower than observed for this case.

Table S5. The cryoprotective agent used for biosystems vitrified in this study.

Biosystem	CPA	Critical cooling rate (CCR) (°C/min)	Critical rewarming rate (CWR) (°C/min)
Coral larvae	10% PG + 5% DMSO + 34% (1 M) trehalose	1.3×10^3	3.5×10^5
Drosophila embryo	27% EG + 9% sorbitol	1.9×10^3	6.8×10^5
Zebrafish embryo	Yolk: 14% (1.8 M) PG + 1.1% (0.7 M) MeOH	3.9×10^5	2.9×10^8
	Outside: 21% (2.7 M) PG + 4% (1.2 M) MeOH + 17.1% (0.5 M) Trehalose	2.0×10^3	6.9×10^5

Critical cooling rate (CCR) and critical rewarming rate (CWR) are calculated based on reference[29].

Table S6. Cryopreservation efficiency of coral larvae.

Method	Larvae per loading	Cooling processing time (mins)	Rewarming processing time (mins)	Target number†	Total time (h)	Sophisticated equipment and training
Manual laser rewarming process	13*	> 3	> 3	100,000	> 17,888.9	Yes
Idealized automated laser rewarming process[30]	13*	> 0.5	> 0.5	100,000	> 94.3	Yes
Cryomesh	200**	< 2	< 2	100,000	< 39.2	No

†Target number refers to the number of viable coral larvae desired after cryopreservation. *Number to achieve the highest direct post rewarming viability is around 43%[31]. **Number is based on a 2 X 2 cm CondD-C (larger mesh sizes are possible and will increase the number accordingly). The cooling and rewarming processing time is based on a single well-trained user of a single Cryotop or cryomesh at one time. The idealized laser rewarming system is based on the laser-associated rewarming method with an automatic handling system (e.g., automated laser alignment and rewarming). This system does not currently exist but is an idealized comparison assuming fully CPA-loaded larvae on a Cryotop that is already cooled. Note that the automatic process assumes the laser is firing at the duty cycle, which is 1 pulse per second during rewarming and is likely an underestimate of the time needed. Finally, it should be noted that no sophisticated equipment is needed for the Cryomesh vs. the laser or automatic process thus making it easily accessible to anyone practicing cryobiology in the field. As coral larvae are chilling sensitive, there are no other reports we are aware of that show success after slow freezing or direct freezing. Laser rewarming is the only other method that has shown success and therefore is used as “conventional” for comparison here.

The cryopreservation efficiency is improved by using CondD-C to achieve high viability and uniform cooling and rewarming with a large number of individual biosystems (i.e., larvae or embryo) loaded (number ≥ 100). As one example, to achieve 100,000 live coral larvae after cryopreservation, the total time of the laser-associated method[31] and an idealized automated laser rewarming system is 456X and 2.4X longer than the cryomesh method, respectively (Table S6). Note, that all coral larvae must be cryopreserved within 2 days, otherwise they will

lose activity and not survive after cryopreservation[31]. CondD-C achieves all of this without the need for any sophisticated equipment or training. Finally, the area / size of the cryomesh area is flexible and scalable and thus can be increased to achieve even faster processing times.

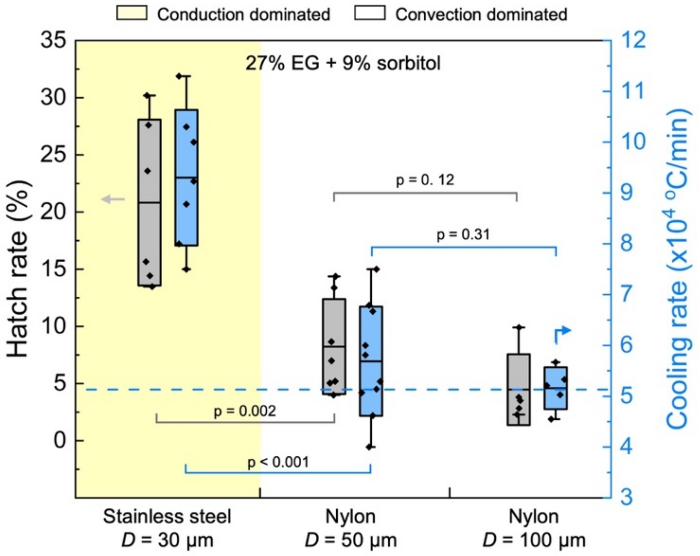

Figure S17. Hatch rate and cooling rate of *Drosophila* embryos on stainless steel and nylon mesh with the vertical plunge. Gray bars are the hatching rate and blue bars are the measured cooling rate of the *Drosophila* embryos. The conduction-dominated cryomesh (yellow-colored area) has a higher hatch rate due to a higher cooling rate.

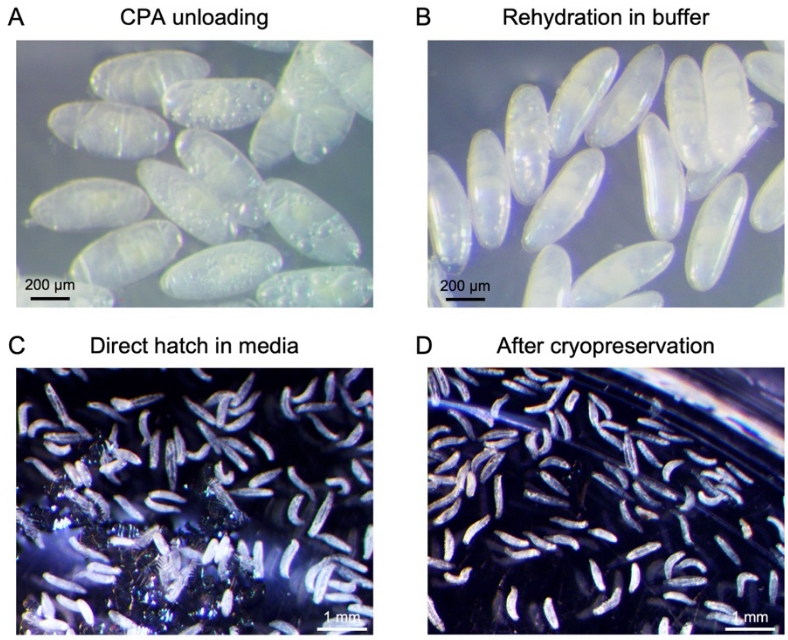

Figure S18. *Drosophila* embryo after cryopreservation. (A) *Drosophila* embryo during CPA unloading process in 15 wt% sucrose solution. (B) *Drosophila* embryo rehydration in cryo buffer. (C) Permeabilized embryos directly hatched in media without cryopreservation. (D) Embryo after cryopreservation hatched in media. We randomly picked 50 coral larvae and 100 *Drosophila* embryos after vitrification and compared those with larvae and embryo without vitrification. The control embryo and larvae morphology showed no change (number of biosystem with morphology change < 1%) compared to those after vitrification and rewarming.

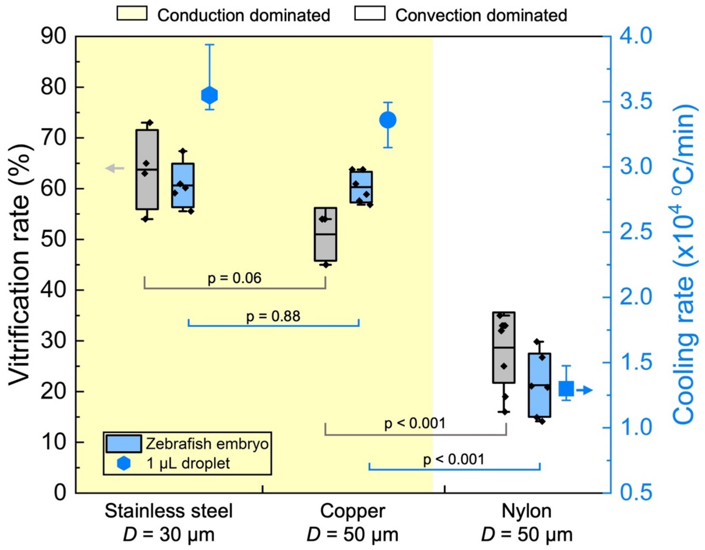

Figure S19. Zebrafish embryo vitrification rate and measured cooling rate on stainless steel, copper, and nylon mesh with $\Phi = 0.5$. Gray bars are the vitrification rate and blue bars are the measured cooling rate of the zebrafish embryo. The copper mesh used has a smaller area ratio (Figure 2C) than the stainless steel mesh, which leads to a lower vitrification rate than stainless steel. This suggests that the cooling of copper mesh with $D = 50$ μm is not as uniform as stainless steel with $D = 30$ μm. Dots are the cooling rate of 1-μL CPA droplet. The conduction-dominated cryomesh (yellow-colored area) has a high vitrification rate due to the high cooling rate.

Table S7. Possible factors leading to decreased viability.

Factors	Required CCR/CWR	Example
CPA non-uniformity inside biosystem	Increase	Fig. 6, CPA inside zebrafish yolk
Decreased contact area	None	Fig. 6, Zebrafish embryo on different mesh
Decreased CPA concentration in biosystem	Increase	Fig. 4, Coral larvae
Biosystem cluster	Increase	Fig. 5, *Drosophila* embryos

Design and physical limits of the cryomesh platform technique

We summarized the design principles for the successful cryopreservation of different biosystems, as well as the physical limits of conduction-dominated cryomesh (Figure S20). To determine how to further improve the cryomesh, we analyzed the achieved and potential viability of biosystems tested in this study (Figure S20). There are three ways to reduce ice crystallization (leading to potential increases in viability): 1) increase the cooling rate, 2) increase CPA concentration, and 3) use more rapid heating source such as Joule heating or laser heating. The coral larvae have the highest viability, up to 85% on CondD-C, with a limit of 100% survival under the best conditions[31]. Thus, the potential for further improvement region based on cooling rate and CPA optimization is limited for coral larvae (shown in the colored area of Figure S20A, with a corresponding increase in survival of approximately 15.2%).

Increasing the cooling rate is the only method considered in this study to improve the survival rate of coral larvae (black arrow, Figure S20A). Meanwhile, the region on the graph that shows potential improvement for the *Drosophila* embryo (highest value of 96%[10]) and zebrafish embryo (highest value of 59%[32]) is 25× and 21× larger, respectively, than for coral larvae, showing the necessity to further improve the cooling rate and CPA in these biosystems. While maintaining the same cooling rate for *Drosophila* embryos, the viability can be improved with a higher CPA concentration to avoid potential ice formation during rewarming (orange dashed line, Figure S20B)[10, 17]. Alternatively, viability can be improved by maintaining the same CPA concentration but with Joule heating as a more rapid heating source (blue dashed line, Figure S20B). For example, improving the cryopreservation protocol to reach the desired viability (yellow star, Figure S20B) requires increasing the cooling rate (along the fitting curve), using a more rapid heating source (blue line), and modifying CPA concentration (orange dashed line). Note, the yellow star is used as an example and does not represent any suggested viability.

The same method to improve viability can be used to design a cryopreservation protocol for zebrafish embryos (Figure S20C). Due to the large size of zebrafish embryos, the theoretical maximum cooling rate is limited to 15.6×10^4 °C/min in the pure conduction case (i.e., assume the surface temperature instantly reaches −196 °C). The potential for cooling improvement is shown as a huge gray-colored area in Figure S20C, demonstrating a large potential for viability improvement. Increasing the cooling rate minimally improves the viability of zebrafish embryos. More rapid heating, used to reach a higher rewarming rate, can improve the viability of zebrafish embryos. More rapid heating can also increase the cryopreservation viability of *Drosophila* embryos with a low CPA concentration, making rapid heating source the most important factor for improvement[17]. In short, the CondD-C achieves the highest viability for small biosystems (thickness < 100 μm) but needs further improvement for large biosystems.

The achievable cooling rate decreases with the increase of biosystem thickness for all different cooling methods (Figure S21A). The dashed lines in Figure S21A show the theoretically maximum cooling rate of different cooling methods. The theoretical cooling rates of cryomesh (blue, orange, and red dashed lines) are calculated based on Equations S5 and S18 with h = 1250 $W/m^2/K$. The purple dashed line is the theoretical limit of the cooling rate by assuming the surface temperature of the biosystem to be −269 °C (temperature of liquid helium). The black dashed line shows the maximum cooling rate that can be achieved with pure conduction heat transfer, assuming the surface temperature of the biosystem to be −196 °C (temperature of LN_2).

We defined three regions among those theoretical cooling rates. The top right corner is the region to be explored with volumetric cooling methods (cooling the entire volume at the same time). The achievable cooling region is for using different cryogens of lower temperature (e.g., liquid helium, −269 °C). The light-blue-colored area is the theoretical cooling rate achieved with conduction heat transfer of biosystem and cryomesh or any other substrates (e.g., cryotop) without consideration of convection heat transfer. In this case, we assumed there was no vapor layer during cooling, which is different from directly printing droplets into LN_2[8]. The gray-colored area shows the cooling rate achieved by convection-dominated cooling methods. Between the conduction cooling and convection cooling regions is the CondD-C cooling method reported in this study, which has a higher cooling rate than convection-dominated cooling and fills the gap between the convection and conduction cooling methods (Figure S21A). The theoretical highest cooling rate of ConvD-C (i.e., nylon cryomesh, orange dashed line, Figure S21A) is still within the convection-dominated region (grey-colored area). By increasing the thermal conductivity of cryomesh, the cooling rate increases and reaches the conduction cooling region. The achievable cooling rate, then, can be determined based on the biosystem thickness for further studies such as CPA optimization. For example, the zebrafish embryo has a thickness of around 350 µm after dehydration. By using CondD-C mesh, the zebrafish embryo can achieve a cooling rate higher than 2.6×10^4 °C/min, which is validated by experimental data (black square, S21A).

Besides cooling rate, CPA concentration is another critical parameter to design the cryopreservation system. A high CPA concentration can be toxic to the biosystem while a low CPA concentration leads to devitrification with ice formation. As a general design principle, a CPA concentration higher than 63.2 wt%[33] is considered toxic to the biosystem (Figure S21B). Then, the lowest CPA concentration required for different biosystem thicknesses is defined with the theoretically maximum cooling rates of different cooling methods (Figure S21B). The CPA concentration (wt%) is calculated based on a well-developed model[29] of PG (propylene glycol). The orange dashed lines present the lowest CPA concentration required to vitrify the biosystem on ConvD-C made of nylon mesh. By increasing the cooling rate of cryomesh (i.e., CondD-C) and avoiding convection heat transfer, the lowest CPA concentration is reduced for a smaller biosystem with a thickness < 200 µm (blue dashed line, Figure S21B). This cryomesh optimal zone is shown by the yellow-colored region between the red and blue dashed lines. The light blue area shows pure conduction cooling by directly printing droplets on cooled plates, which can achieve no CPA (pure water) vitrification[6]. The area below the purple dashed line

will lead to ice formation, even using pure conduction methods, due to low CPA concentration. For a general cryopreservation design on cryomesh, the initial CPA concentration tests can be chosen from the yellow-colored region using CondD-C. Then, the optimal CPA concentration can be increased to facilitate successful vitrification based on the cryomesh optimal zone.

Like the cooling rate, for all different warming methods, the achievable rewarming rate also decreases with the increase of biosystem thickness for all different rewarming methods (Figure S22A). The dashed lines show the theoretical maximum cooling rate of different cooling methods. The theoretical rewarming rates of cryomesh (blue, orange, and red dashed lines) are calculated based on Equation S5 with h = 5000 W/m^2/K. The purple dashed line is the theoretical limit of the rewarming rate by using the Joule heating[17]. We defined three regions among those theoretical limits of rewarming rates. The top right corner is the region to be explored with volumetric rewarming methods such as laser rewarming[34]. The light magenta area shows the achievable rewarming rate, which can be further improved based on CondD-C. The gray-colored area shows the rewarming rate achieved by convection-dominated methods. The theoretical highest rewarming rate of ConvD-C (i.e., nylon cryomesh, orange dashed line, Figure S22A) is still within the convection-dominated region (gray area). Similar to the cooling rate, by increasing the thermal conductivity of cryomesh, the maximum rewarming rate increases. Because of the correlation between the cryomesh's conductivity and the rewarming rate, the achievable rewarming rate can be defined based on the biosystem thickness for further studies, such as studies CPA optimization. For example, these coral larvae have a thickness of around 100 μm. By using CondD-C mesh, coral larvae can achieve a rewarming rate higher than 1×10^5 °C/min (higher than the red dashed line), which is validated by experimental data (blue square, S22A).

The lowest CPA concentration required for different biosystem thicknesses is determined using the theoretical maximum rewarming rates of different cooling methods (Figure S22B). The CPA concentration (wt%) is calculated based on a well-developed model[29] of PG (propylene glycol). The orange dashed lines show the lowest CPA concentration required to vitrify the biosystem on ConvD-C of nylon mesh. By increasing the thermal conductivity of cryomesh (i.e., CondD-C), the lowest CPA concentration is reduced for a smaller biosystem with a thickness < 200 μm (blue dashed line, Figure S22B). Thus, the yellow-colored region between the red and blue dashed lines is defined as the cryomesh optimal zone. The area below the purple dashed line will lead to ice formation due to low CPA concentration even using Joule heating. For general cryopreservation design on cryomesh, the initial CPA concentration tests

can be chosen from the yellow-colored region with CondD-C. Then, the optimal CPA concentration can be increased to facilitate successful vitrification based on the cryomesh optimal zone. A higher CPA concentration can help avoid ice formation but should be optimized to avoid toxicity to the biosystem.

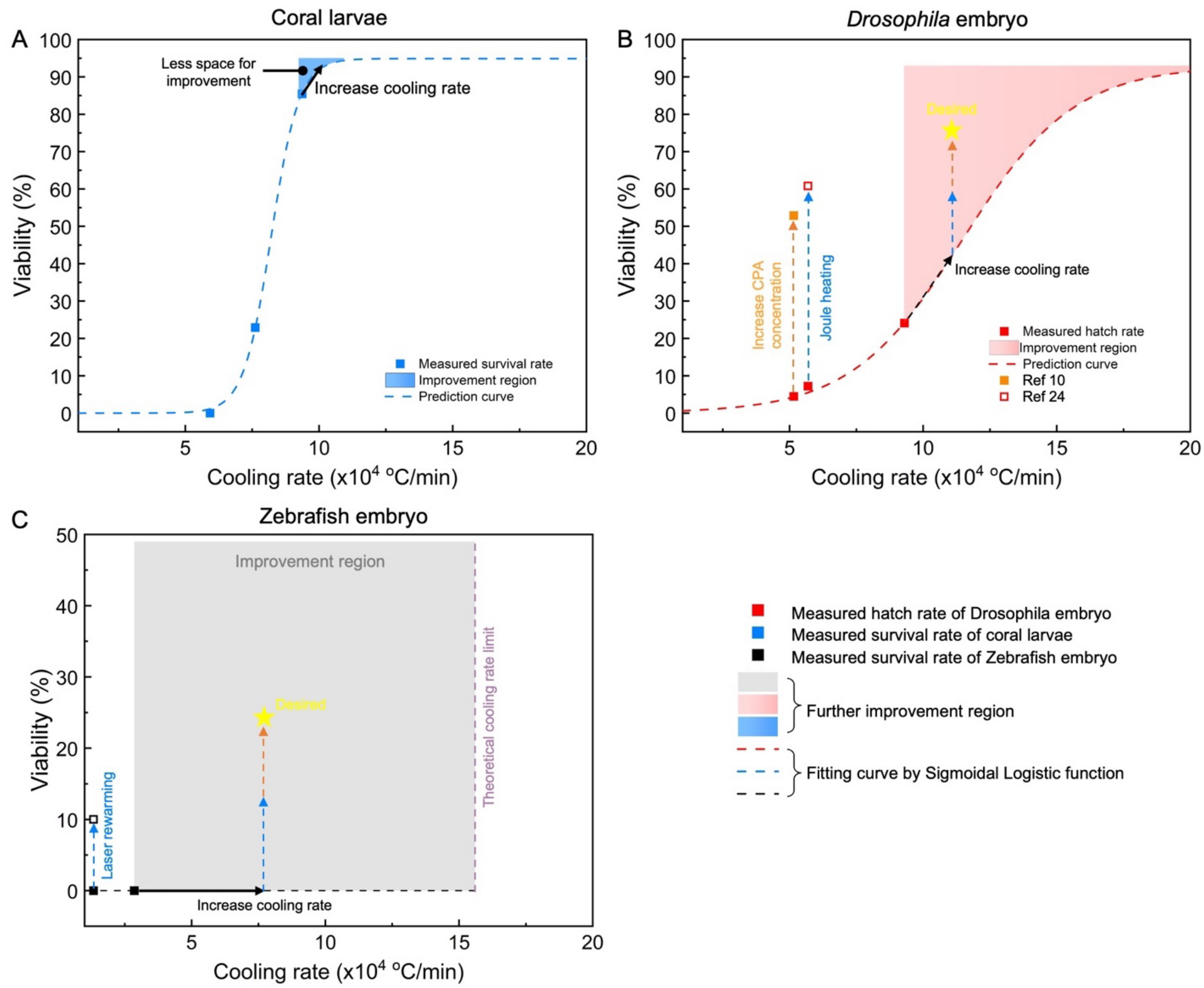

Figure S20. A general guide to further improve the viability of biosystems used in this study. (A) Potential for improvement for coral larvae is limited. (B) Potential improvement of the viability of *Drosophila* embryo. (C) Potential improvement of the viability of zebrafish embryos. Viability of the biosystem increases with the increase in cooling rate. The colored region shows the potential improvement in the viability of different biosystems by increasing the cooling rate and optimizing CPA concentration. The dashed lines show predicted viability increasing with cooling rate for three biosystems. The yellow star shows one example of desired viability.

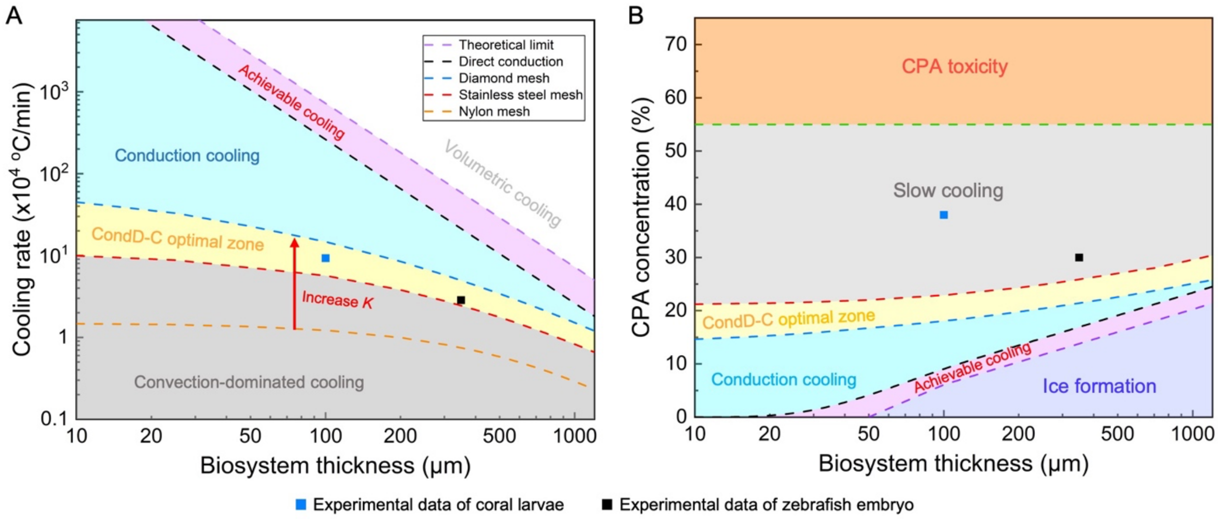

Figure S21. Design and physical limits of cryomesh cooling. (A) Increasing biosystem thickness reduces the achievable cooling rate in the biosystem. The dashed lines show the highest cooling rate achieved by different mesh materials and different cooling methods. (B) Selection of cryoprotective agent (CPA) concentration (wt%) with different cooling methods. The upper boundary of the cryomesh optimal zone (red dashed line) is the lowest CPA concentration required for CondD-C. The lower boundary of the cryomesh optimal zone (blue dashed line) is the lowest CPA concentration required for the diamond mesh (CondD-C). CPA toxicity is the major failure mode of cryopreservation, shown in the top, orange region.

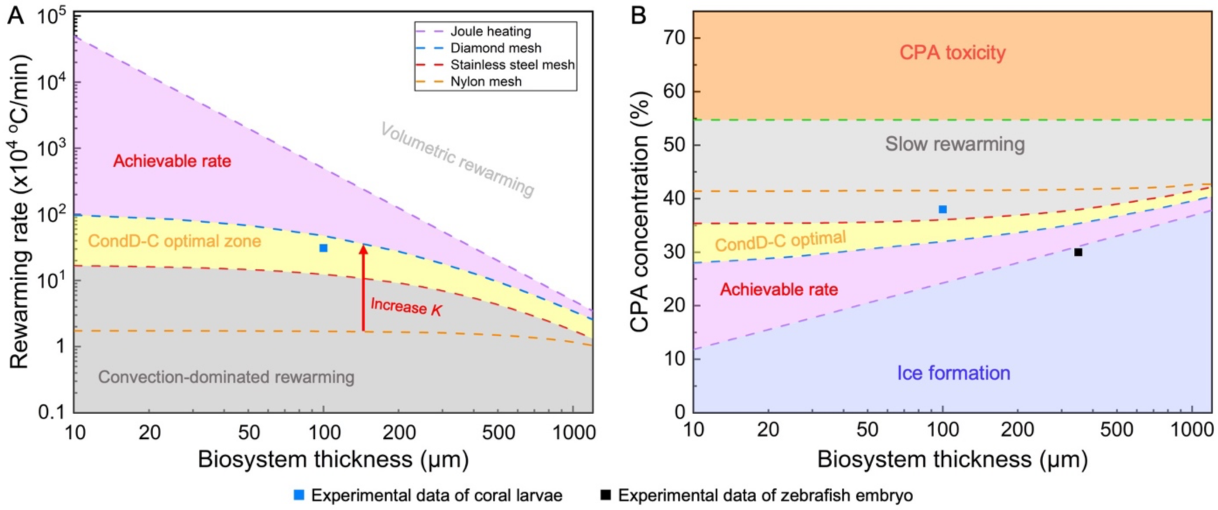

Figure S22. Design and physical limits of cryomesh rewarming. (A) Increased biosystem thickness reduces the achievable rewarming rate in the biosystem. The dashed lines show the highest rewarming rate achieved by different mesh materials and different cooling methods. (B) Selection of cryoprotective agent (CPA) concentration (wt%) with different rewarming methods.

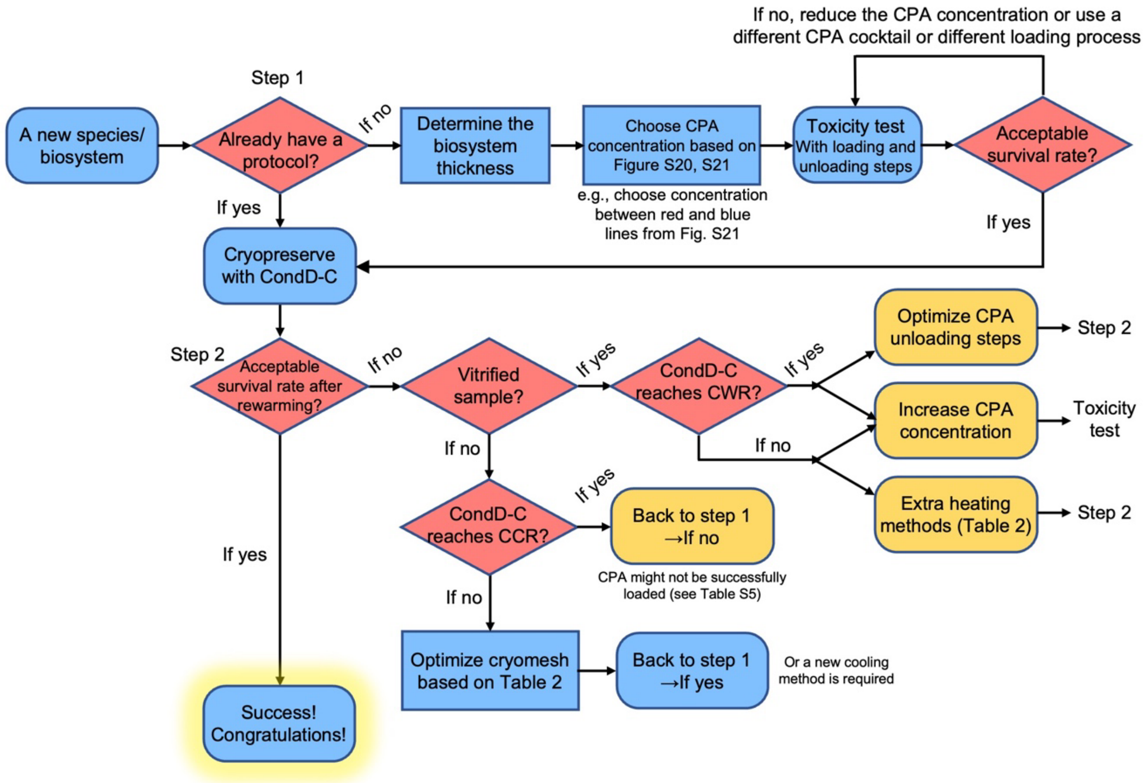

Figure S23. General flowchart to use CondD-C for cryopreservation. The optimization of cryomesh and more rapid heating methods can be found in Table 2. For a thick biosystem (thickness > 300 μm), more rapid heating is recommended to achieve a higher rewarming rate than the CWR.

Table S8. Table of mesh physical properties.

Material	Hydrophobicity (contact angle °)*	Young's modulus (GPa)[35]	Fracture toughness ($MPa \cdot m^{1/2}$)	Adhesion rate** (%)	Wash-off rate*** (%)	Potential Toxicity
Nylon	Hydrophilic (73°)	2-4	5-10[36]	> 99	> 99	No
Stainless steel	Hydrophilic (71°)	180	112-278[37]	> 99	> 99	No
Copper	Hydrophilic (86°)	117	80-100[38]	> 99	> 99	Yes

*Hydrophobicity is determined on the plain surface without any structure or treatment, which determines the wicking performance. Hydrophobicity of the bulk material is reflective of the relative performance of potential mesh materials. The contact angle has a standard deviation of ± 5°. **Adhesion rate is defined as the ratio of the number of biosystems (e.g., Drosophila embryos) attached to the mesh after the LN_2 plunging process / the total number of the biosystems initially loaded onto the mesh. (Figure S24A and S24B). ***Wash-off rate is defined as the ratio of the number of biosystems released from mesh after rewarming/unloading / the total number of biosystems attached to the mesh prior to rewarming (Figure S24C and S24D). Adhesion and wash-off rates were measured for coral larvae, *Drosophila* embryos, and Zebrafish embryos, using counts from images taken before and after the relevant processing steps. Example images for Drosophila are shown in Figure S24. Rates for coral larvae on the copper mesh were not analyzed due to the identified toxicity issues.

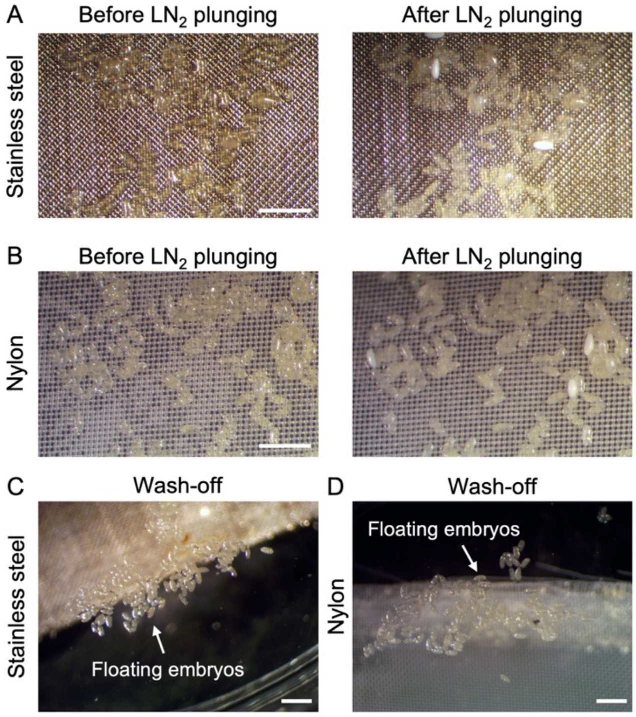

Figure S24. Drosophila embryo adhesion rate on (A) stainless steel and (B) nylon. Drosophila embryo wash-off rate of (C) Stainless steel and (D) nylon. The scale bars are 1 mm.

To improve the performance of the cryomesh, we considered additional parameters for design and modification (Table S8). Hydrophilic (contact angle < 90°) cryomesh is preferred because it facilitates rapid nitrogen bubble release and enhanced wicking of excess CPA. Hydrophilic mesh has a high surface energy, which allows the LN_2 to wet the cryomesh easily[39]. Thus, the bubbles generated by boiling have a small contact area on and can easily be released during plunge cooling. The reduced bubble wrapping increases the heat transfer between cryomesh and LN_2. When the cryomesh is wetted with CPA (after loading the biosystem with CPA on mesh), a meniscus will form in between wires due to the surface tension force[40]. Hydrophilic wires will lead to a smaller contact angle between the meniscus and the wires, which generates a concave shape due to capillary pressure[41]. Thus, the CPA has the potential to wet through the mesh pore and more easily wick off. Finally, surface hydrophobicity will also impact the adhesion rate and wash-off rate of different meshes, which is an important performance factor. We defined the adhesion rate of the number of biosystems (e.g., embryos or larvae) attached to the mesh after the LN_2 plunging process / the total number of the biosystems initially loaded onto the mesh. Wash-off rate shows the number of biosystems released from the mesh after rewarming and unloading / the total number of biosystems attached to the mesh prior to rewarming. A gentle pipetting can also be applied to help the biosystem release during unloading. A high adhesion rate (> 90%) is desired to reduce the loss of the biosystem during vitrification. Hydrophilicity will enhance the adhesion rate by generating a high surface tension force. Meanwhile, a high wash-off rate (> 90%) ensures all the cryopreserved biosystems can be collected after vitrification and rewarming. Adhesion and wash-off rates were measured for coral larvae, *Drosophila* embryos, and Zebrafish embryos, using counts from images taken before and after the relevant processing steps. For all the cases analyzed, adhesion and wash-off rates were >99% (Table S8 and Figure S24).

Besides the consideration of the heat transfer performance of cryomesh, the consideration of mechanical properties can further enhance the cryopreservation performance. As a practical consideration, we also included information on the materials' relative strength. A high Young's modulus may not be required for larger mesh wire diameters (e.g., ≥ 50 μm) but is required for smaller wires to avoid breaking the mesh due to loading, handling, and surface tension of CPA. A high fracture toughness (K_{IC}) is also beneficial to withstand potential thermal stresses that can accumulate during rapid cooling and rewarming of the mesh [42, 43]. Similarly, materials with a fracture toughness ≤ 1 are not recommended as a practical design, such as glass.

To improve the performance of the cryomesh, we considered additional parameters for design and modification (Table S8). Hydrophilic (contact angle < 90°) cryomesh is preferred because it facilitates rapid nitrogen bubble release and enhanced wicking of excess CPA. Hydrophilic mesh has a high surface energy, which allows the LN_2 to wet the cryomesh easily[39]. Thus, the bubbles generated by boiling have a small contact area on and can easily be released during plunge cooling. The reduced bubble wrapping increases the heat transfer between cryomesh and LN_2. When the cryomesh is wetted with CPA (after loading the biosystem with CPA on mesh), a meniscus will form in between wires due to the surface tension force[40]. Hydrophilic wires will lead to a smaller contact angle between the meniscus and the wires, which generates a concave shape due to capillary pressure[41]. Thus, the CPA has the potential to wet through the mesh pore and more easily wick off. Finally, surface hydrophobicity will also impact the adhesion rate and wash-off rate of different meshes, which is an important performance factor. We defined the adhesion rate of the number of biosystem (e.g., embryos or larvae) attached to the mesh after the LN_2 plunging process / the total number of the biosystem initially loaded onto the mesh. Wash-off rate shows the number of biosystem released from the mesh after rewarming and unloading / the total number of biosystem attached to the mesh prior to rewarming. A gentle pipetting can also be applied to help the biosystem release during unloading. A high adhesion rate (> 90%) is desired to reduce the loss of the biosystem during vitrification. Hydrophilicity will enhance the adhesion rate by generating a high surface tension force. Meanwhile, a high wash-off rate (> 90%) ensures all the cryopreserved biosystems can be collected after vitrification and rewarming. *Drosophila* embryos were used as a model system to test the adhesion rate and wash-off rate on stainless steel and nylon mesh (Figure S24). The adhesion rate and wash-off rate of copper mesh were also tested with the *Drosophila* embryo. As copper mesh was toxic and we will need to modify the coating, data of coated copper mesh were not included in this manuscript. One possible coating to address copper toxicity could be PEG (polyethylene glycol)[44].

Besides the consideration of the heat transfer performance of cryomesh, the consideration of mechanical properties can further enhance the cryopreservation performance. As a practical consideration, we also included information on the materials' relative strength. A high Young's modulus may not be required for larger mesh wire diameters (e.g., ≥ 50 μm) but is required for smaller wires to avoid breaking the mesh due to loading, handling, and surface tension of CPA. A high fracture toughness (K_{IC}) is also beneficial to withstand potential thermal stresses that can accumulate during rapid cooling and rewarming of the mesh[42, 43]. Similarly, materials with

a fracture toughness ≤ 1 are not recommended as a practical design, such as glass. With the consideration of the heat transfer performance of cryomesh, the consideration of mechanical properties can further enhance the cryopreservation performance.

Biocompatibility of the cryomesh is another concern as some materials may lead to potential toxicity to the biosystem. The goal of this work was to provide design principles to optimize the heat transfer of the cryomesh and then provide proof-of-concept using model organism systems. Thus, we used a preliminary biocompatibility assessment of different meshes based on the morphology of the coral larvae[31] and hatching for *Drosophila* embryo[10]. We found copper to be toxic to the coral larvae, whereas stainless steel and plastic meshes did not show toxicity. For the *Drosophila* embryo, all tested cryomesh showed no toxicity to the embryo. It should also be noted that even the toxicity observed for the copper mesh could potentially be addressed by further surface modifications. Future studies will be needed to investigate the biocompatibility of cryomesh materials and coatings more comprehensively with assessments such as cytotoxicity, proliferation, and potential immune responses.

Besides the biocompatibility of cryomesh, the long-term effect of cryopreservation will be further investigated. Previous study observed that coral larvae produced with frozen sperm are indistinguishable from their non-cryopreserved counterparts (same genotypes, etc.) in their development, settlement rates, and uptake of their symbionts[45]. Coral sperm frozen for up to 10 years produced coral offspring and was useful in assisted gene flow[46]. These corals are still alive 5 years later. Cryopreserved zebrafish embryos that were laser-warmed developed and produced normal offspring[32]. For *Drosophila* embryos, fertility and SNP after cryopreservation can be retained across multiple generations. No lethal mutations were introduced after cryopreservation or long-term LN_2 storage[10]. It is clear that, after cryopreservation, fish, *Drosophila*, and coral, can survive and continue to thrive. Future studies will conduct a more comprehensive analysis of the long-term effect and potential damage of cryopreservation.

References:

[1] Y. Liu, X. Xu, X. Ma, E. Martin‐Rendon, S. Watt, Z. Cui, *Biotechnol. Prog.* **2010**, *26*, 1635.
[2] J. Baboo, P. Kilbride, M. Delahaye, S. Milne, F. Fonseca, M. Blanco, J. Meneghel, A. Nancekievill, N. Gaddum, G. J. Morris, *Sci. Rep.* **2019**, *9*, 1.
[3] L. Rienzi, C. Gracia, R. Maggiulli, A. R. LaBarbera, D. J. Kaser, F. M. Ubaldi, S. Vanderpoel, C. Racowsky, *Hum. Reprod. Update* **2017**, *23*, 139.
[4] Cryotop cooling rate https://www.kitazato-ivf.com/vitrification/cryotop/, accessed: 11/05, **2022**.
[5] K. Mochida, A. Hasegawa, D. Shikata, N. Itami, M. Hada, N. Watanabe, T. Tomishima, A. Ogura, *Sci. Rep.* **2021**, *11*, 1.
[6] Y. Akiyama, M. Shinose, H. Watanabe, S. Yamada, Y. Kanda, *Proc. Natl. Acad. Sci. U.S.A.* **2019**, *116*, 7738.
[7] M. Shi, K. Ling, K. W. Yong, Y. Li, S. Feng, X. Zhang, B. Pingguan-Murphy, T. J. Lu, F. Xu, *Sci. Rep.* **2015**, *5*, 1.
[8] L. Zhan, S. Z. Guo, J. Kangas, Q. Shao, M. Shiao, K. Khosla, W. C. Low, M. C. McAlpine, J. Bischof, *Adv. Sci.* **2021**, *8*, 2004605.
[9] L. Zhan, J. S. Rao, N. Sethia, M. Q. Slama, Z. Han, D. Tobolt, M. Etheridge, Q. P. Peterson, C. S. Dutcher, J. C. Bischof, *Nat. Med.* **2022**, *28*, 798.
[10] L. Zhan, M.-g. Li, T. Hays, J. Bischof, *Nat. Commun.* **2021**, *12*, 1.
[11] F. P. Incropera, D. P. DeWitt, T. L. Bergman, A. S. Lavine, *Fundamentals of heat and mass transfer*, Wiley New York, **1996**.
[12] Y. Zhang, G. Zhao, S. C. Hossain, X. He, *Int. J. Heat Mass Transf.* **2017**, *114*, 1.
[13] H. Hu, Y. Sun, *J. Appl. Phys.* **2012**, *112*.
[14] F. P. Incropera, A. S. Lavine, T. L. Bergman, D. P. DeWitt, *Fundamentals of heat and mass transfer*, Wiley, **2007**.
[15] The Engineering ToolBox (2011). Metals, Metallic Elements and Alloys - Thermal Conductivities. [online] Available at: https://www.engineeringtoolbox.com/thermal-conductivity-metals-d_858.html accessed: 09/08, **2023**.
[16] Z. Han, A. Sharma, Z. Gao, T. W. Carlson, M. G. O'Sullivan, E. B. Finger, J. C. Bischof, *Adv. Healthc. Mater.* **2020**, *9*, 2000796.
[17] L. Zhan, Z. Han, Q. Shao, M. L. Etheridge, T. Hays, J. C. Bischof, *Nat. Commun.* **2022**, *13*, 1.
[18] E. Brentari, P. J. Giarratano, *Boiling heat transfer for oxygen, nitrogen, hydrogen, and helium*, US National Bureau of Standards, **1965**.
[19] J. Park, S. Woo, S. Kim, M. Kim, W. Hwang, *ACS omega* **2019**, *4*, 18304.
[20] Y. Ye, S. Klimchuk, M. Shang, K. McDonald, J. Niu, *ACS Appl. Mater. Interfaces* **2019**, *11*, 16944.
[21] H. Cha, H. Vahabi, A. Wu, S. Chavan, M.-K. Kim, S. Sett, S. A. Bosch, W. Wang, A. K. Kota, N. Miljkovic, *Sci. Adv.* **2020**, *6*, eaax0746.
[22] H. Jo, H. S. Park, M. H. Kim, *Int. J. Heat Mass Transf.* **2016**, *93*, 554.
[23] D. I. Shim, G. Choi, N. Lee, T. Kim, B. S. Kim, H. H. Cho, *ACS Appl. Mater. Interfaces* **2017**, *9*, 17595.
[24] R. Iwata, L. Zhang, K. L. Wilke, S. Gong, M. He, B. M. Gallant, E. N. Wang, *Joule* **2021**, *5*, 887.
[25] J. Li, D. Kang, K. Fazle Rabbi, W. Fu, X. Yan, X. Fang, L. Fan, N. Miljkovic, *Sci. Adv.* **2021**, *7*, eabg4537.
[26] Y. Song, C. D. Díaz‐Marín, L. Zhang, H. Cha, Y. Zhao, E. N. Wang, *Adv. Mater.* **2022**, *34*, 2200899.
[27] M. M. Rahman, E. Olceroglu, M. McCarthy, *Langmuir* **2014**, *30*, 11225.

[28] M. Jiang, Y. Wang, F. Liu, H. Du, Y. Li, H. Zhang, S. To, S. Wang, C. Pan, J. Yu, *Nature* **2022**, *601*, 568.
[29] J. Kangas, L. Zhan, Y. Liu, H. Natesan, K. Khosla, J. Bischof, *J. Heat Transf.* **2022**, *144*, 031207.
[30] S. B. Kodandaramaiah, D. M. Gohl, A. Alegria, A. S. Joshi, B. Auch (US Patent), US20220309705A1, **2022**.
[31] J. Daly, N. Zuchowicz, C. I. Nuñez Lendo, K. Khosla, C. Lager, E. M. Henley, J. Bischof, F. W. Kleinhans, C. Lin, E. C. Peters, *Sci. Rep.* **2018**, *8*, 1.
[32] K. Khosla, J. Kangas, Y. Liu, L. Zhan, J. Daly, M. Hagedorn, J. Bischof, *Adv. Biosyst.* **2020**, *4*, 2000138.
[33] Z. Han, L. Gangwar, E. Magnuson, M. Etheridge, C. O. Pringle, J. Bischof, J. Choi, *Cryobiology* **2022**, *106*, 113.
[34] K. Khosla, K. Smith, J. Kangas, L. Gangwar, A. Joshi, Y. Liu, G. Han, M. McAlpine, S. Kodandaramiah, M. Hagedorn, *Cryobiology* **2021**, *103*, 174.
[35] The Engineering ToolBox (2003). Young's Modulus, Tensile Strength and Yield Strength Values for some Materials. [online] Available at: https://www.engineeringtoolbox.com/young-modulus-d_417.html, accessed: 09/08, **2023**.
[36] G. E. Dieter, *ASM Handbook, Volume 20-Materials Selection and Design*, ASM international, **1997**.
[37] R. A. Lula, *Toughness of Ferritic Stainless Steels*, American Society for Testing and Materials, **1980**.
[38] M. F. Ashby, D. CEBON, *Le Journal de Physique IV* **1993**, *3*, C7.
[39] L. Zhang, S. Gong, Z. Lu, P. Cheng, E. N. Wang, *Int. J. Heat Mass Transf.* **2022**, *182*, 121904.
[40] A. Tuteja, W. Choi, M. Ma, J. M. Mabry, S. A. Mazzella, G. C. Rutledge, G. H. McKinley, R. E. Cohen, *Science* **2007**, *318*, 1618.
[41] T. L. Liu, C.-J. C. Kim, *Science* **2014**, *346*, 1096.
[42] Z. Huang, S. Zhang, R. Yang, X. Wu, R. Li, H. Zhang, P. Hung, *Fuel* **2020**, *266*, 117040.
[43] P. K. Solanki, J. C. Bischof, Y. Rabin, *Cryobiology* **2017**, *76*, 129.
[44] Z. Gao, H. L. Ring, A. Sharma, B. Namsrai, N. Tran, E. B. Finger, M. Garwood, C. L. Haynes, J. C. Bischof, *Adv. Sci.* **2020**, *7*, 1901624.
[45] M. Hagedorn, V. L. Carter, E. M. Henley, M. J. Van Oppen, R. Hobbs, R. E. Spindler, *Sci. Rep.* **2017**, *7*, 14432.
[46] M. Hagedorn, C. A. Page, K. L. O'Neil, D. M. Flores, L. Tichy, T. Conn, V. F. Chamberland, C. Lager, N. Zuchowicz, K. Lohr, *Proc. Natl. Acad. Sci. U.S.A.* **2021**, *118*, e2110559118.
